# Identifying rare genetic variants in 21 highly multiplex autism families: the role of diagnosis and autistic traits

**DOI:** 10.1038/s41380-022-01938-4

**Published:** 2023-01-26

**Authors:** Ravi Prabhakar More, Varun Warrier, Helena Brunel, Clara Buckingham, Paula Smith, Carrie Allison, Rosemary Holt, Charles R. Bradshaw, Simon Baron-Cohen

**Affiliations:** 1 Autism Research Centre, Department of Psychiatry, University of Cambridge, Cambridge, United Kingdom; 2 Gurdon Institute, University of Cambridge, Cambridge, United Kingdom

**Keywords:** Genetics, Autism spectrum disorders

## Abstract

Autism is a highly heritable, heterogeneous, neurodevelopmental condition. Large-scale genetic studies, predominantly focussing on simplex families and clinical diagnoses of autism have identified hundreds of genes associated with autism. Yet, the contribution of these classes of genes to multiplex families and autistic traits still warrants investigation. Here, we conducted whole-genome sequencing of 21 highly multiplex autism families, with at least three autistic individuals in each family, to prioritise genes associated with autism. Using a combination of both autistic traits and clinical diagnosis of autism, we identify rare variants in genes associated with autism, and related neurodevelopmental conditions in multiple families. We identify a modest excess of these variants in autistic individuals compared to individuals without an autism diagnosis. Finally, we identify a convergence of the genes identified in molecular pathways related to development and neurogenesis. In sum, our analysis provides initial evidence to demonstrate the value of integrating autism diagnosis and autistic traits to prioritise genes.

## Introduction

Autism is a group of neurodevelopmental conditions characterised by difficulties communicating socially in addition to engaging in unusually restricted and repetitive behaviours and sensory difficulties. Autism is substantially heritable with a prevalence of 0.9–2.6%, with twin and familial heritability ranging from 60 to 90% [1, 2]. Variants across the allelic spectrum are associated with autism: de novo protein-truncating and missense variants in highly constrained genes [3, 4] at one end of the allelic frequency spectrum and common single nucleotide polymorphisms at the other end [5]. Considerable progress has been made in identifying genes, with information largely coming from de novo protein-truncating variants in highly constrained genes [3, 4, 6, 7]. However, de novo variants together account for less than approximately 3% of the total attributable variance [3, 8].

Most of the existing evidence comes from the study of simplex (one autistic individual per family) families, and recent studies have identified the role of rare inherited variation in autism. For instance, Simons Simplex Collection data shows that autistic individuals have a higher frequency of private, inherited truncating single nucleotide variants (SNVs) and copy number variants (CNVs) compared to non-autistic siblings [9]. The results of another study indicated that families with multiple autistic individuals (multiplex families) are enriched for both inherited and de novo rare CNVs [10]. Finally, a recent study identified an excess of inherited protein-truncating and missense variation in constrained genes in multiplex families [9, 11]. Rare inherited variants, missense and Protein Truncating Variant (PTV) together account for up to 6% of the total likelihood of autism [8, 9]. Given that having a family relative with an autism diagnosis substantially increases an individual’s likelihood of autism [1], studying the genetics of autism using highly multiplex families may aid in identifying genes associated with autism. Furthermore, given that family relatives of autistic individuals have elevated scores on measures of subclinical autism features - termed autistic traits [12] - incorporating information from measures of autistic traits may further aid in identifying genes associated with autism. Recent studies have investigated the effects of rare variants (inherited and de novo) associated with autism on autistic traits [13–15], but to our knowledge, no study has integrated autistic trait information with autism diagnosis to prioritise genetic variants.

To understand the contribution of rare inherited single-nucleotide variants (SNVs), Insertions/Deletions (INDELs) and de novo variants in highly multiplex autism families, we conducted Whole Genome Sequencing (WGS) on 21 families with three or more autistic individuals in the immediate family. We collected information on autistic traits as measured using the Autism Spectrum Quotient [16] (adult version or age equivalent measure for those under 16 years of age) for all individuals. We first investigated the association between de novo and inherited rare variants in autistic individuals. We then incorporated information regarding autistic traits where individuals were classified into subgroups based on their autistic traits, and investigated the relative contribution of de novo and inherited rare variants in these groups.

## Materials and methods

### Participants

We recruited 21 highly multiplex families into the study (*n* = 112 participants). We define highly multiplex families as families with three or more individuals with an autism diagnosis in the immediate family. These families were recruited between 2014 to 2018. Ten families had four or more individuals in the immediate family with an autism diagnosis. For one family (Family 10), we were unable to recruit all autistic individuals with an autism diagnosis and thus had genetic information only on two autistic members in the family. In total, 50 of the 112 individuals were females, and 76 had a diagnosis of autism (34 autistic females) from a qualified professional. Four individuals (three females) were suspected to have an autism diagnosis but had not been formally assessed at the time of participation in the study. Diagnosis of autism was provided by qualified clinicians independent of the study team. The study team obtained copies of the diagnostic report to verify the assessment of the autism diagnosis. Pedigree diagrams of all families are provided in Supplementary Fig. 1.

All participants completed a measure of autistic traits: The adult Autism Spectrum Quotient [16], the adolescent Autism Spectrum Quotient [17], the child Autism Spectrum Quotient [18], or the Quantitative Checklist for Autism in Toddlers [19]. The details of autistic trait information are provided in Supplementary Fig. 2. All participants provide one or more samples of saliva using Oragene-500 saliva kit. DNA was quantified and extracted by LGC using protocols designed to extract DNA from Oragene-500 saliva kits (GEN-9300-330).

The study obtained ethical approval from the Human Biology Research Ethics Committee (HBREC.2015.02). Informed consent was obtained from all participants.

### Whole genome sequencing and quality control

For all samples, Whole Genome Sequencing was done using the Illumina HiSeq 4000 platform with paired-end reads of 150 base pairs. In read preprocessing, reads with low quality (less than Q20) were filtered out and low quality bases were trimmed from the ends of the reads. Adaptors were removed from both pairs using the Trim Galore tool (https://zenodo.org/badge/latestdoi/62039322). Shorter fragments where the reads overlapped were joined to provide fragments of >150 bases [20]. After pre-processing, the filtered data were aligned to the human genome reference assembly GRh37/hg19 using BWA-MEM 0.7.12 [21]. Next, PCR duplicates were removed using Picard tools (https://broadinstitute.github.io/picard/) and Base Quality Score Recalibration, as well as INDEL realignment, was performed using GATK 3.7 [22]. Using the Qualimap 2 tool, summary statistics were obtained to assess the effectiveness of read mapping and alignment quality mainly the total proportion of mapped reads and coverage of the reference genome [23].

Single-nucleotide polymorphism (SNPs) and Insertions/Deletions (INDELs) were called using GATK 4.1 Haplotypecaller [22]. Finally, the called SNPs and INDELs were annotated with the Annovar tool [24, 25]. We retained rare non-synonymous variants (minor allele frequency <1% according to the Non-Finnish European (NFE) population from the Genome Aggregation Database (gnomAD) [26] along with variants considered to be pathogenic by SIFT [27] and PolyPhen-2 [28] algorithms, stop-gain and stop-loss variants. For disruptive INDELs, we considered frameshift insertion/deletion, stop-gain, and stop-loss variants. All SNP/INDELs variants were supported by at least 10 reads, and heterozygous variants had at least 33% reads corresponding to the alternate allele. A separate variant filtering analysis was performed for each of the 21 families for autistic traits and diagnosis approaches.

To identify de novo variants in trios (father, mother, and child), we analysed 44 trios identified in 9 of the 21 families. De novo variant calling was performed using the VarScan2 [29] trio option with parameters (minimal coverage 10, minimal variant frequency 0.20, *p*-value 0.05, adjacent variant frequency 0.05, and adjacent *p*-value 0.15). For the variant filtering step, we considered the following main criteria; 1) Reads depth greater than 20 in all three samples; 2) Alternate allele reads greater than or equal to 8 in the child; 3) Heterozygous de novo status of the variant in the child with alternate allele frequencies greater than 40% and absent in parents (alternate allele frequency less than 5%); 4) Non-synonymous and rare variants less than or equal to 1% in gnomAD NFE population; 5) Disruptive variants were considered based on SNV (Non-synonymous, SIFT and PolyPhen2 HDIV), frameshift In/Dels, stop gain/loss, and splice junctions. The resultant variants encoding genes are prioritised based on pLI greater than or equal to 0.9 and considered for the gene candidates.

### Identifying and prioritising inherited and de novo variants within a family

Given that autism may be underdiagnosed in older individuals [30], we used information from both autism diagnosis and autistic traits to identify potential genetic variants in the pedigree. For autistic phenotypic traits, we divided individuals into four groups based on their scores on the Autism Spectrum Quotient (AQ), which was developed in a previous study of over 3000 individuals: [31] 1) Broad Autistic Phenotype (BAP) (between 1 - 2 standard deviations from the mean AQ score); 2) Medium Autistic Phenotype (MAP, 2 - 3 standard deviations from the mean); 3) Narrow Autistic Phenotype (NAP, 3+ standard deviations from the mean); and 4) Average or low autistic traits, which encompassed all other participants [31]. In the variant filtering process, we considered variants based on their presence in phenotypic traits in the family: 1) BAP variants were present in individuals of three groups (BAP, MAP, and NAP); 2) MAP variants were present in two groups (MAP and NAP); 3) NAP variants were present in only in individuals from the NAP group. The total number of individuals considered for the filtering criteria for autistic traits in each family is given in Supplementary Table 1. In the diagnosis-level variant filtering approach, we prioritised variants based on the autistic diagnosis of individuals in the pedigree. For this purpose, we applied two main criteria; 1) The variant had to be shared among at least two individuals diagnosed with autism; 2) The variant had to be absent in family members who were not autistic. We used the same criteria to identify variants associated with autistic traits. Specifically, for each autistic trait subgroup, the variant had to be present in at least two individuals in that autistic trait subgroup, or a higher autistic trait subgroup, and must be absent from other individuals. Variant prioritisation for each family was done using autistic traits in all groups and diagnosis status using bespoke scripts available at: https://github.com/ravimore8386/VarFilter-PD. We restricted our analyses to variants in constrained genes identified by using two related metrics: a probability of loss-of-function intolerance score greater than 0.9, and a LOEUF in the first decile (less than 0.37) set of genes from gnomAD.

### Identifying candidate genes in published studies and databases

**Fig. 1:**
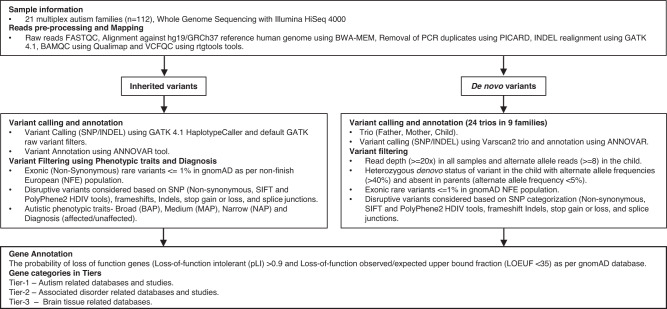
Overview of the rare inherited SNP/INDEL and de novo variants analysis pipeline used in this study. The separate variant calling and filtering were conducted for de novo and inherited variants. After this, variants were grouped into three tiers. SNV single nucleotide variant, Indel insertion or deletion, NFE Non-Finish European, GATK The Genome Analysis Toolkit, pLI Loss-of-function intolerant, LOEUF Loss-of-function observed/expected upper bound fraction, SIFT Sorting Intolerant From Tolerant, PolyPhen-2 Polymorphism Phenotyping v2, BAP Broad Autistic Phenotype, MAP Medium Autistic Phenotype, NAP Narrow Autistic Phenotype.

Based on previous studies and existing databases, we curated four groups of genes associated with autism, co-occurring conditions, and human brain tissue. First, to identify genes that are robustly associated with autism or other severe neurodevelopmental conditions (Tier 1), we investigated whether they are present in one of the following databases: SFARI Gene (Categories S, 1–3) [32], SPARK Gene [33], Satterstrom et al., 2020 (99 genes) [3], De Rubeis et al., (2014) 107 genes [34], DDG2P developmental disorders database (monoallelic confirmed, probable, or possible) [35], or Developmental Brain Disorder Gene Database (DBDGD) (https://dbd.geisingeradmi.org/) [36]. Tier 2 consists of genes with less robust evidence linking them to autism, intellectual disability, or co-occurring conditions such as epilepsy, and ADHD. In Tier 2, we included genes with evidence from the following databases: AutDB (1225 genes) [37], Gene4Denovo (106 genes) [38], autismKB (171 genes) [39], epilepsy gene-sets of EpilepsyGene (499 genes) [40] and Wang et al., 2017 (621 genes) [41], Intellectual Disability databases IDGenetics (496 genes) [42] and sysID (1224 genes) [43], and ADHDgene (359 genes) [44]. Finally, we defined a third tier (Tier 3), which consists of brain tissue-related genes present in the databases Allen Brain Atlas (1000 genes) [45] and The Human Protein Atlas (1460 genes) [46], SynaptomeDB (1886 genes) [47]. Figure 1 shows the flowchart of the analysis performed in this study.

### Analysis of the overrepresentation of genes

The genes prioritised in Tiers 1-3 from inherited and denovo analysis were inputted into PANTHER (Protein ANalysis THrough Evolutionary Relationships) to perform gene set overrepresentation and enrichment analysis for specific molecular functions, biological processes, cellular components, and protein class [48]. Protein-protein interaction networks were predicted using the STRING database (STRING 168 v9.1; http://string-db.org/) and only interactions with a high confidence score (≥0.7) were considered [49].

## Results

Overall, 84% of the WGS reads from saliva samples could be mapped to the reference human genome hg19 with 90.05% of bases at a coverage of at least 20x for 112 individuals. From this data, approximately 4,144,188 variants per individual were called with a transitions (Ti) to transversions (Tv) ratio of 2.02 to 2.03. On average, we detected 24,704 exonic SNPs per individual (synonymous: 12,448; nonsynonymous: 12,253). For INDELs the average was 1,045,875 variants per individual; 423 Frameshifts; 37 Stop gains/losses, and 136 Splicing variants. Detailed variant statistics are given in Supplementary Fig. 2.

### De novo variants in constrained genes

As a class, protein-truncating de novo variants in constrained genes are strongly linked to autism [3, 4]. Therefore, we initially focussed on this class of variants in 10 families where de novo variant calling was possible. After variant filtering, we identified six variants in six constrained genes (*DYNC1H1, KDM3A, ZBTB18, TAF4, DGKI*, and *PHLPP1*) with pLI > 0.9, which were detected in seven autistic individuals from six families. Three of these genes (*DYNC1H1*, *KMD3A*, and *ZBTB18*) have been robustly associated with autism [3] and severe developmental disorders (results presented in Table 1). Furthermore, *TAF4* has been previously associated with autism [33, 50] and a missense mutation in *TAF4* (A124P) was identified in two autistic individuals from two different families (Families 4 and 21) in our study. Despite observing this twice in our datasets, it is not observed in gnomAD non-Finnish European (NFE) populations. This is in accordance with previous studies that demonstrate the enrichment of ultra-rare variants (i.e., variants that are not observed in the ExAC/gnomAD population database) in autism [4, 51]. Finally, we also identified a frameshift deletion variant in *PHLPP1*, a gene with known structural variants (18q21.2) that have been associated with intellectual disability [52] but not with autism.

**Table 1:** Summary of rare genetic variants encoding genes that are either inherited or de novo.

Gene symbol	Gene name	Inherited/de novo	Family	Trait/Diagnosis	Chr: Start	Ref/Alt	AA change	dbSNP	MAF NFE	SIFT	P2 HDIV	Notes	Tier
*KMT2C*	Lysine Methyltransferase 2C	Inherited	19	MAP	7:151932996	C/T	p.G892E	rs372408170	0.0001	D	D	DDG2P mono-allelic probable	1
*ANKRD11*	Ankyrin Repeat Domain 11	Inherited	10	NAP/Diagnosis	16:89346774	G/T	p.P2059H	rs117997391	0.0075	D	D	DDG2P mono-allelic confirmed	1
*SETD2*	SET Domain Containing 2, Histone Lysine Methyltransferase	Inherited	14	MAP/Diagnosis	3:47165380	G/A	p.S205F	.	.	D	P	DDG2P mono-allelic probable	1
			10	NAP/Diagnosis	3:47164711	C/T	p.R428H	rs201984344	0.0004	D	D		
*ATP2B2*	ATPase Plasma Membrane Ca2+ Transporting 2	Inherited	15	BAP	3:10392216	G/A	p.R683C	rs754680596	.	D	D	SFARI gene 2	1
*CUX1*	Cut Like Homeobox 1	Inherited	16	MAP/Diagnosis	7:101921292	A/C	p.K530Q	rs118010189	0.0071	D	D	DDG2P possible	1
*JADE2*	Jagged Canonical Notch Ligand 1	Inherited	14	NAP	5:133901944	C/T	p.R370C	rs137957798	0.0005	D	D		2
*MTOR*	Mechanistic Target Of Rapamycin Kinase	Inherited	25	NAP	1:11272478	T/C	p.Y1151C	rs151082401	0.0003	D	D	DDG2P mono-allelic confirmed	1
*BRPF1*	Bromodomain And PHD Finger Containing 1	Inherited	10	NAP/Diagnosis	3:9783082	G/A	p.V605M	.	.	D	P	DDG2P mono-allelic confirmed	1
*ESR1*	Estrogen Receptor 1	Inherited	10	NAP/Diagnosis	6:152265352	C/T	p.R269C	rs142712646	0.0008	D	D		2
*MAP2*	Microtubule Associated Protein 2	Inherited	16	MAP/Diagnosis	2:210559050	G/A	p.G719D	rs148922251	0.0051	D	D		2
*SEMA3F*	Semaphorin 3F	Inherited	22	MAP	3:50225538	C/T	p.P684L	rs754678639	.	D	D		2
*YTHDC1*	YTH Domain Containing 1	Inherited	28	BAP/Diagnosis	4:69203301	C/A	p.D150Y	rs186920853	.	D	D	DBDGDB Score 2	1
*CHD7*	chromodomain helicase DNA binding protein 7	Inherited	19	Diagnosis	8:61707624	G/T	p.D726Y	rs748119797	.	D	D	DDG2P mono-allelic confirmed	1
*SON*	SON DNA and RNA binding protein	Inherited	19	Diagnosis	21:34923617	A/G	p.T694A	rs141608426	0.0034	D	D	DDG2P mono-allelic confirmed	1
*PREX1*	Phosphatidylinositol-3,4,5-trisphosphate dependent Rac exchange factor 1	Inherited	3	Diagnosis	20:47309282	C/T	p.G322S	rs202212653	.	D	D	SFARI gene 2	1
*PTPRT*	Protein tyrosine phosphatase receptor type T	Inherited	10	Diagnosis	20:40735430	G/C	p.T1129R	rs201830301	6.48E-05	D	D	SFARI gene 3	1
*RIMS2*	Regulating synaptic membrane exocytosis 2	Inherited	21	Diagnosis	8:104897660	C/T	p.S86F	rs17854256	0.0062	D	P	DDG2P mono-allelic probable	1
*KDM5A*	Lysine demethylase 5A	Inherited	4	Diagnosis	12:430192	G/A	p.P837L	rs745469846	6.48E-05	D	D	DDG2P possible	1
*DYNC1H1*	Dynein cytoplasmic 1 heavy chain 1	de novo	15	BAP/Diagnosis	14:102509021	C/T	p.P4150L	.	.	D	D	DDG2P mono-allelic confirmed	1
*KDM3A*	Lysine demethylase 3A	de novo	4	MAP/Diagnosis	2:86716780	A/G	p.K1191E	.	.	D	D	DBDGDB Score 3	1
*ZBTB18*	zinc finger and BTB domain containing 18	de novo	22	N /Diagnosis	1:244217437	G/C	p.V112L	.	0	D	D	DDG2P mono-allelic confirmed	1
*TAF4*	TATA-box binding protein associated factor 4	de novo	4/21	MAP/BAP/Diagnosis	20:60640497	C/G	p.A124P	.	.	D	P		2
*CLOCK*	Clock Circadian Regulator	Inherited	22	BAP	4:56319253	C/G	p.E392Q	rs373421741	.	D	P		2
*JAG1*	Jagged Canonical Notch Ligand 1	Inherited	19	MAP	20:10621462	T/G	p.R1056S	rs146006022	0	D	P		2
*NCOR2*	Nuclear Receptor Corepressor 2	Inherited	10	NAP/Diagnosis	12:124832388	C/T	p.R1342H	rs36081651	0.0066	D	D		2
*STXBP5L*	Syntaxin Binding Protein 5 Like	Inherited	15	BAP	3:120952486	G/A	p.V379M	rs61996323	0.0084	D	D		2
*TNS3*	Tensin 3	Inherited	15	BAP	7:47331594	G/A	p.T1296M	rs41280696	0.0086	D	D		2
*TTC28*	Tetratricopeptide Repeat Domain 28	Inherited	14	NAP	22:28426233	G/A	p.R1352C	rs201500299	7.00E-04	D	D		2
*PTCH1*	patched 1	Inherited	19	Diagnosis	9:98242733	G/A	p.P295L	rs370755364	.	D	D	DBDGDB Score 3	1
*PHLPP1*	PH domain and leucine rich repeat protein phosphatase 1	de novo	7	NAP/Diagnosis	18:60506115	C/-	p.Y624fs	.	.	NA	NA		2
*CASKIN1*	CASK Interacting Protein 1	Inherited	19	MAP/Diagnosis	16:2231252	G/A	p.S706L	rs201599923	3.00E-04	D	D		3
*WDR7*	WD Repeat Domain 7	Inherited	10	NAP/Diagnosis	18:54547385	C/G	p.A1139G	rs773660280	.	D	D	DDG2P probable	1
*FAT3*	FAT Atypical Cadherin 3	Inherited	14	MAP/Diagnosis	11:92534043	G/T	p.V2622F	rs17615477	0.0054	D	D		3
*MACF1*	Microtubule Actin Crosslinking Factor 1	Inherited	3	NAP/Diagnosis	1:39853641	G/T	p.A2981S	rs144760259	0.0052	D	D	DDG2P mono-allelic probable	1
		Inherited	10	NAP/Diagnosis	1:39763365	G/T	p.C815F	rs148207245	0.0069	D	P	DDG2P mono-allelic probable	1
*PCDH1*	Protocadherin 1	Inherited	15	BAP	5:141243507	G/A	p.R418C	rs143703336	6.48E-05	D	D		3
*VCAN*	Versican	Inherited	14	NAP	5:82786147	G/T	p.V101L	rs758097509	.	D	D		3
*PCLO*	Piccolo presynaptic cytomatrix protein	Inherited	11	Diagnosis	7:82584378	G/A	p.T1964M	rs148432464	0	D	P		3
*PSMD1*	Proteasome 26S subunit, non-ATPase 1	Inherited	19	Diagnosis	2:232010978	A/G	p.M675V	rs201118764	0	D	D	SFARI 1	1
*TP53BP1*	Tumour protein p53 binding protein 1	Inherited	22	Diagnosis	15:43714134	C/A	p.G1340V	rs570488146	.	D	P		3
*DGKI*	Diacylglycerol kinase iota	de novo	4	BAP/Diagnosis	7:137531385	C/A	p.G75V	.	.	D	P		3

*Chr* chromosome, *Start* start chromosomal position according to hg19/GRCh37, *Ref* reference allele, *Alt* alternative allele, *AA change* alteration on the protein level, *MAF NFE* minor allele frequency in Non-Finish European (NFE) population as per GnomAD database, *P2* HDIV-PolyPhen-2, *HDIV* HumDiv-trained, *D* damaging/probably damaging/deleterious/disease-causing, *P* possibly damaging, *NA* not applicable.

### Segregation of inherited variants based on autistic traits and diagnoses within families

We then focused on inherited variants, primarily focusing on individuals with an autism diagnosis. We examined rare variants in constrained genes that are shared by at least two diagnosed autistic individuals in a family but are absent in all non-autistic individuals in the same family. Using this approach, we identified a total of 37 variants encoding 35 genes (Table 1). Fifteen of these genes have previously been linked to either autism or severe undiagnosed developmental disorders. Examples of these genes include *CHD7, ANKRD11*, and *SON* which are robustly associated with both autism and severe developmental disorders. In addition, we identified three genes with less robust evidence linking them to autism or other co-occurring conditions (Tier 2), and four further genes enriched in brain tissue.

Given that many individuals with autism may be undiagnosed, especially in older generations, we further expanded our analyses to include traits associated with autism. We defined three groups for the autistic traits (NAP, MAP, and BAP; see the Methods section for more details) using self- or parent-reported measures of autistic traits (in the case of children). This classification was done agnostic to an autism diagnosis, but based on autistic trait scores. This also allowed for the classification of family members who had not sought a diagnosis for various reasons. It resulted in 47 individuals in the NAP group, 21 in the MAP group, 25 in the BAP group, and the remaining were outside of these three groups.

Based on the analysis of autistic traits, we found three additional genes with robust evidence for association with autism and severe developmental disorders. This includes a rare missense variant in *MTOR*, that is shared by individuals in the NAP group including individuals without an autism diagnosis. We also identified seven additional Tier 2 genes and two additional Tier 3 genes (Table 1). This illustrates the potential utility of considering a trait-based approach in addition to a diagnosis-based approach for gene discovery.

**Fig. 2:**
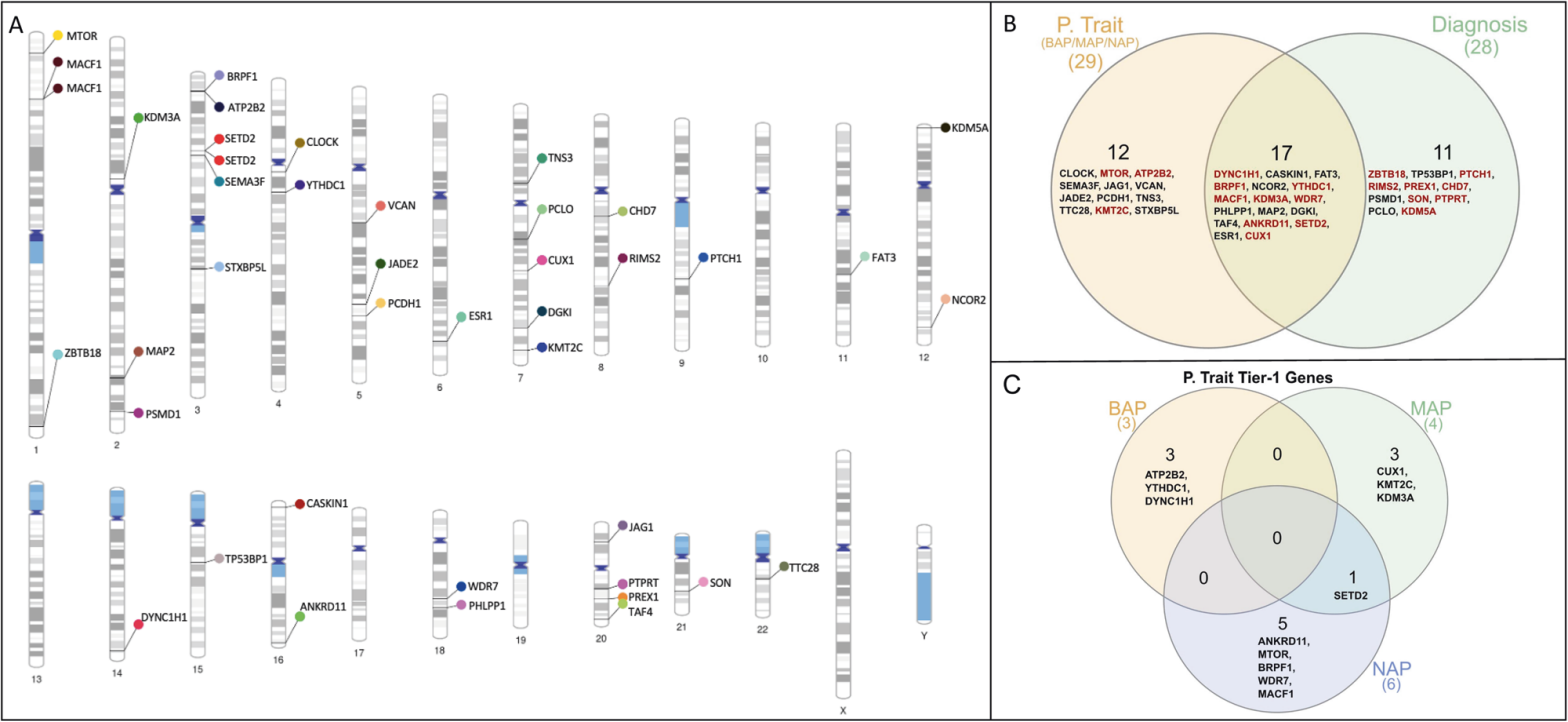
Identified prioritised genes in the phenotype trait and diagnosis approaches in multiplex autism families. **A** Variants harbouring genes across all chromosomes. **B** Venn diagram showing the common and unique genes identified in phenotypic traits and diagnosis. Tier-1 genes are highlighted in red colour. **C** The distribution of the Tier 1 genes identified in BAP, MAP, and NAP phenotypic trait categories.

All the prioritised genes (de novo and inherited variants) obtained from both approaches (phenotypic traits and diagnosis) were plotted across chromosomes using PhenoGram (Fig. 2A). We identified a total of 17 genes that are implicated using both approaches (phenotypic traits and diagnosis), 12 were identified only using the trait-based approach, and 11 were identified only using the diagnosis-based approach (Fig. 2B). Of the 17 genes implicated using both approaches, nine (*ANKRD11, SETD2, CUX1, BRPF1, YTHDC1, DYNC1H1, KDM3A, WDR7*, and *MACF1*) are Tier 1 genes and are highlighted in red. Of the 11 identified only using the diagnosis-based approach, eight were Tier 1 (*CHD7, SON, PREX1, PTPRT, RIMS2, KDM5A, PTCH1, ZBTB18* and *PSMD1*). In contrast, only three Tier 1 genes (*KMT2C, ATP2B2*, and *MTOR*) were identified in the phenotypic trait-based approach. The distribution of the Tier 1 genes identified in the phenotypic trait-based (BAP, MAP, and NAP) are depicted in Fig. 2C. Six genes were identified in the NAP group, four in the MAP group, and three in the BAP group. This suggests that a greater number of Tier 1 genes are identified with increasing stringency of the AQ cut-offs.

Whilst the above two approaches highlight genes based on sharing, we also investigated how many individuals with an autism diagnosis were carriers of rare variants in a Tier 1 gene. In total, 50 out of the 74 individuals with autism diagnosis were carriers of rare variants in a Tier 1 gene. In comparison, only 11 of the 34 individuals without an autism diagnosis and who did not suspect they were undiagnosed autistic, were carriers of a rare variant in a Tier 1 gene ( *χ*^2^ (1, *N* = 108) = 11.75, *p* = 6.07 ×10^−4^). Altogether, this suggests that autistic individuals are enriched for rare genetic variants in genes previously linked to autism and/or neurodevelopmental conditions.

### Analysis of functional and gene ontology (GO) terms

We next conducted functional and gene ontology (GO) analysis using genes in the three Tiers, to identify shared molecular processes. We identified significant enrichment in terms pertaining to developmental and neuronal processes (Table 2).

**Table 2:** A list of overrepresented functional categories based on GO enrichment analysis of genes.

Gene ontology	GO term	Fold enrichment	*p*-value	False discovery rate (FDR)	#Genes	Genes symbol
Biological process	Sex differentiation (GO:0007548)	6.78	2.83E−04	4.51E−02	7	*PKD1, KDM5A, TAF4, ESR1, CHD7, PATZ1*
	Regulation of neurogenesis (GO:0050767)	6.78	2.60E−05	9.54E−03	8	*MAP2, SEMA3F, MTOR, RELA, MACF1, CUX1, CHD7, STK11*,
	Regulation of developmental growth (GO:0048638)	6.57	1.04E−04	2.29E−02	7	*MAP2, SEMA3F, PTCH1, MTOR, RIMS2, MACF1, CHD7*
	Regulation of neuron projection development (GO:0010975)	6.13	1.72E−05	7.13E−03	9	*MAP2, PREX1, FAT3, PKD1, SEMA3F, MTOR, MACF1, CUX1, STK11*
	Developmental process involved in reproduction (GO:0003006)	4.37	3.18E−06	1.86E−03	14	*KDM3A, PKD1, SETD2, PTCH1, KDM5A, TAF4, MTOR, BPTF, CLOCK, ESR1, CHD7, PATZ1, STK11, ATP2B2*
	Positive regulation of developmental process (GO:0051094)	3.32	6.69E−05	1.70E−02	14	*PREX1, ACIN1, PKD1, PTCH1, SP1, TP53BP1, MTOR, JAG1, RELA, RIMS2, MACF1, CUX1, CHD7, STK11*
Molecular function	Transcription regulatory region sequence-specific DNA binding (GO:0000976)	2.93	0.000144	0.0457	15	*SP1, KDM5A, TAF4, MTOR, ZBTB18, BPTF, RELA, CLOCK, BAZ2A, NCOR2, HIC2, CUX1, ESR1, CHD7, PATZ1*
Cellular component	Presynaptic cytoskeleton (GO:0099569)	66.33	0.000593	0.0514	2	*PCLO, RIMS2*
	Presynaptic active zone (GO:0048786)	15.92	0.000142	0.0177	4	*PCLO, DGKI, RIMS2, ATP2B2*
	Dendritic growth cone (GO:0044294)	66.33	0.000593	0.0493	2	*MAP2, PTCH1*
	Glutamatergic synapse (GO:0098978)	7.83	2.51E−06	0.000717	9	ATP2B2, PTPRT, SEMA3F, PCLO, MTOR, DGKI, ACAN, RELA, ATP2B2

**Fig. 3:**
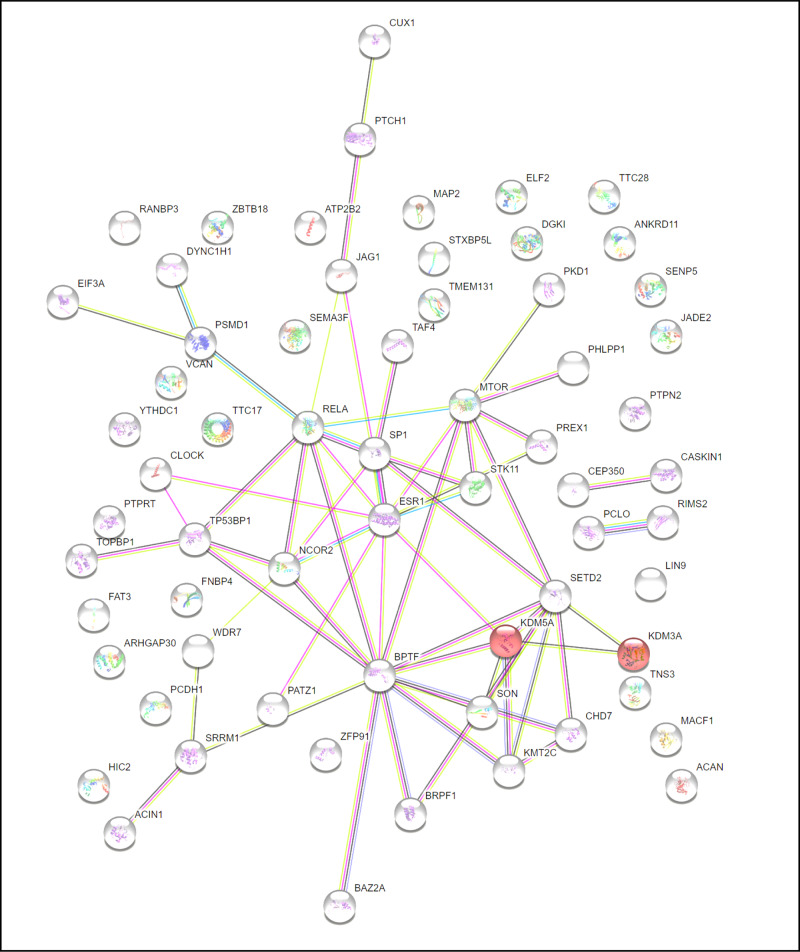
Protein–protein interaction (PPI) Gene network analysis based on the STRING database of prioritised genes in pedigrees. Protein–protein interaction (PPI) analysis was conducted using all genes identified in the current study across all tiers (both inherited and de novo).

Additionally, we used the STRING database to search for protein-protein interactions, as shown in Fig. 3. Analysis showed that *KDM5A* and *KDM3A* strongly interact and co-express together in the HDMs demethylate histones (HSA-3214842) pathway (FDR 0.0345). Interestingly, we found an inherited SNP variant (rs745469846) harboured in *KDM5A* and a de novo SNP variant (chr2:86716780) in the *KDM3A* gene in Family 4. Histone demethylation genes have previously been implicated in autism [53], and these variants suggest that perturbation of their function may result in the autism phenotype.

## Discussion

In this study, we conducted whole-genome sequencing on 21 highly multiplex autism families and used both autistic trait and diagnosis status to identify potential genetic variants associated with autism. Our study highlights the value of autistic traits in identifying potential genetic variants associated with autism.

We first focused on genes with robust evidence linking them to autism or severe developmental conditions (Tier 1 genes) to identify molecular genetic diagnosis in these 21 families. In individuals where de novo variant calling was possible, we identified three autistic individuals who had a rare, protein-altering variant in highly constrained genes previously implicated in autism or severe developmental conditions, suggesting that even in multiplex families, de novo variants may be a genetic contributor to autism. For rare, inherited variants, we used a different approach. We focussed on genes shared in at least two autistic individuals and absent in any individual without an autism diagnosis. Overall, the approach identified rare variants in twenty different genes known to be associated with autism or other developmental conditions.

Two genes (SETD2 and MACF1) were identified in two separate families. Both genes are associated with both diagnosis and high autistic traits (NAP and MAP) and are classified as Tier 1 genes. Rare, protein altering genetic variants in *SETD2* were identified in MAP and NAP individuals in two families, indicating variants in this gene may increase autistic traits. *SETD2* is on chromosome 3, and codes for the SET domain containing 2 protein. This protein is a histone methyltransferase that trimethylates lysine 36 of histone H3 in nucleosomes. Genetic variants in *SETD2* are associated with autism [54]. According to van Rij et al., (2018) [55], de novo frameshift mutations were found in the *SETD2* gene in two people with Luscan-Lumish syndrome, who were diagnosed with intellectual disability, speech delay, macrocephaly, facial dysmorphism, and autism. Additionally, protein altering variants in *MACF1* (Microtubule-actin crosslinking factor 1) were found in NAP individuals in two families. The *MACF1* gene is known to be involved in multiple neural processes during development, including neurite outgrowth and neuronal migration [56].

Focusing again on rare variants in Tier 1 genes, we identified a modest excess of this class of variants in individuals with an autism diagnosis compared to those without an autism diagnosis. Approximately two-thirds of all autistic individuals in our study carried a rare variant in a Tier 1 gene. Whilst we are unable to provide a genetic diagnosis in this study, this excess of rare variants in autistic individuals suggests a genetically complex aetiology.

Finally, we conducted pathway analyses to identify convergence at the level of biological mechanisms, using genes identified across all three tiers. This analysis highlighted the role of both neuronal development and synaptic activity in the genes that we have identified so far, which is in line with biological mechanisms implicated by previous studies of genes associated with autism.

Previous studies have investigated the effects of genes associated with autism on autistic traits. However, these studies have not integrated autistic traits and autism diagnosis to prioritise genes, possibly because: 1) a large number of participants in existing cohorts come from simplex families (e.g., Simon’s Simplex Collection); 2) these cohorts are not consistently measured for autistic traits; and 3) autistic traits measures are predominantly used for children with almost none of the large cohorts having used the same measure of autistic traits in both children and parents, making comparisons difficult. In our study, we were not statistically well-powered to identify new genes, and so investigated if rare protein altering variants in constrained genes already linked to autism, related conditions, or highly expressed in the brain are observed individuals with a diagnoses or individuals with high autistic traits. We identified individuals without an autism diagnosis who are carriers of protein altering rare variants in constrained genes previously implicated in autism. Whilst autistic traits measure are not a proxy for undiagnosed autism, this approach highlights the value of using trait-based information in addition to clinical diagnosis. This approach may be particularly relevant for older individuals in families with diagnosed autistic individuals, as they may be autistic but not have a clinical diagnosis.

This is one of the largest WGS studies focussing exclusively on highly multiplex autism families, however, this study does have some limitations. Whilst WGS does help to identify genetic variants, the current study is not statistically well-powered to identify novel genes. However, our approach of dividing genes into tiers of evidence helps highlight potential candidate genes that may reach statistical significance using larger sample sizes. In addition, the ability to measure autistic traits in our study demonstrates that at least some individuals without an autism diagnosis are carriers of rare variants in genes that have been implicated in autism. Nevertheless, these findings need to be validated and extended in other cohorts.

To this end, there is a need to collect measures of autistic traits in parents, harmonise different measures of autistic traits using different approaches (e.g., factor analysis), and investigate to what extent these findings are generalisable to different family types (e.g., families with just one or two individuals who have an autism diagnosis). Whilst we use the AQ in the current study, it is unclear to what extent these findings are generalisable to other measures of autistic traits. Furthermore, recent studies have demonstrated that autistic traits are multi-dimensional [14, 57], so future research needs to identify whether specific dimensions of autistic traits are enriched for different genetic variants.

Regarding different family types, previous research has indicated that both the genetic architecture of autism and the phenotypic variation in autistic traits differ between simplex and multiplex families. Compared to multiplex families, autistic individuals have enriched for de novo protein-truncating variants in constrained genes [11, 58], and non-autistic family members have scores on measures of autistic traits closer to the general population [59]. It is unclear to what extent our findings are relevant for simplex families, who are over-represented in large autism cohorts. Finally, we did not have further phenotypic information such as motor coordination, adaptive behaviour, or intellectual functioning of the participants of this study. Therefore, we were unable to investigate if these factors also contribute.

In conclusion, we report the results of whole-genome sequencing of 21 highly multiplex autism families. By combining both autistic trait and diagnostic information, we identify several rare, protein altering variants in constrained genes linked to autism or related conditions in these families. We demonstrate a modest excess of rare variants in genes associated with autism and/or neurodevelopmental conditions in autistic individuals compared to non-autistic family members. Our study demonstrates the value of combining both diagnosis and trait-based information to prioritise candidate genes for further investigation.

### Supplementary information

Supplementary legends

Supplementary Fig. 1

Supplementary Fig. 2

Supplementary Table 1

## Data Availability

Variant-level genetic and phenotypic data for this study from participants who have consented to share the data are available from the European Genome-phenome Archive (EGA; https://www.ebi.ac.uk/ega/).
