# Supplementary material for: Identifying rare genetic variants in 21 highly multiplex autism families: the role of diagnosis and autistic traits
Source: Mol Psychiatry. 2023 Jan 26;28(5):2148–57. doi: 10.1038/s41380-022-01938-4 (PMC10575770; doi:10.1038/s41380-022-01938-4)
Supplement: Supplementary file 1 — Supplementary legends [file 41380_2022_1938_MOESM1_ESM.docx]

**Supplementary** **legends**

Supplementary Fig. 1: Detailed information about the pedigrees of families selected for genome sequencing from autism-affected families.

Supplementary Fig. 2: The details of phenotype and diagnosis information of family and sample-wise variant profiles contain a total of SNPs/INDELs, nonsynonymous SNPs, and disruptive INDELs.

Supplementary Table 1: The total number of individuals in the family and the filtering criteria used for phenotype-based variant prioritization.
