# Supplementary material for: Identifying rare genetic variants in 21 highly multiplex autism families: the role of diagnosis and autistic traits
Source: Mol Psychiatry. 2023 Jan 26;28(5):2148–57. doi: 10.1038/s41380-022-01938-4 (PMC10575770; doi:10.1038/s41380-022-01938-4)
Supplement: Supplementary file 2 — Supplementary Fig. 1 [file 41380_2022_1938_MOESM2_ESM.pdf]

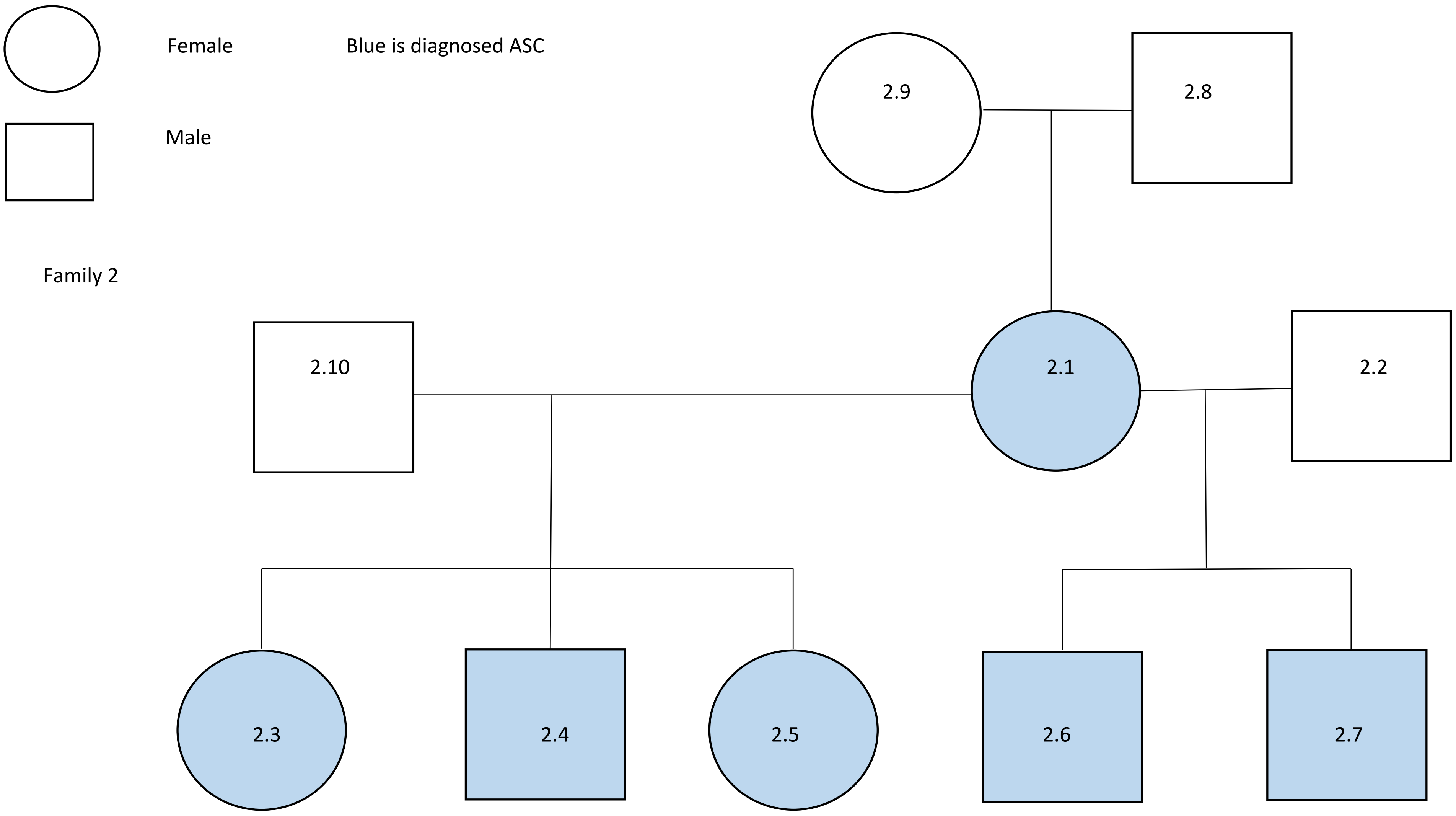
Female
Blue is diagnosed ASC
Male
Family 2
2.9
2.8
2.10
2.1
2.2
2.3
2.4
2.5
2.6
2.7

Family 3

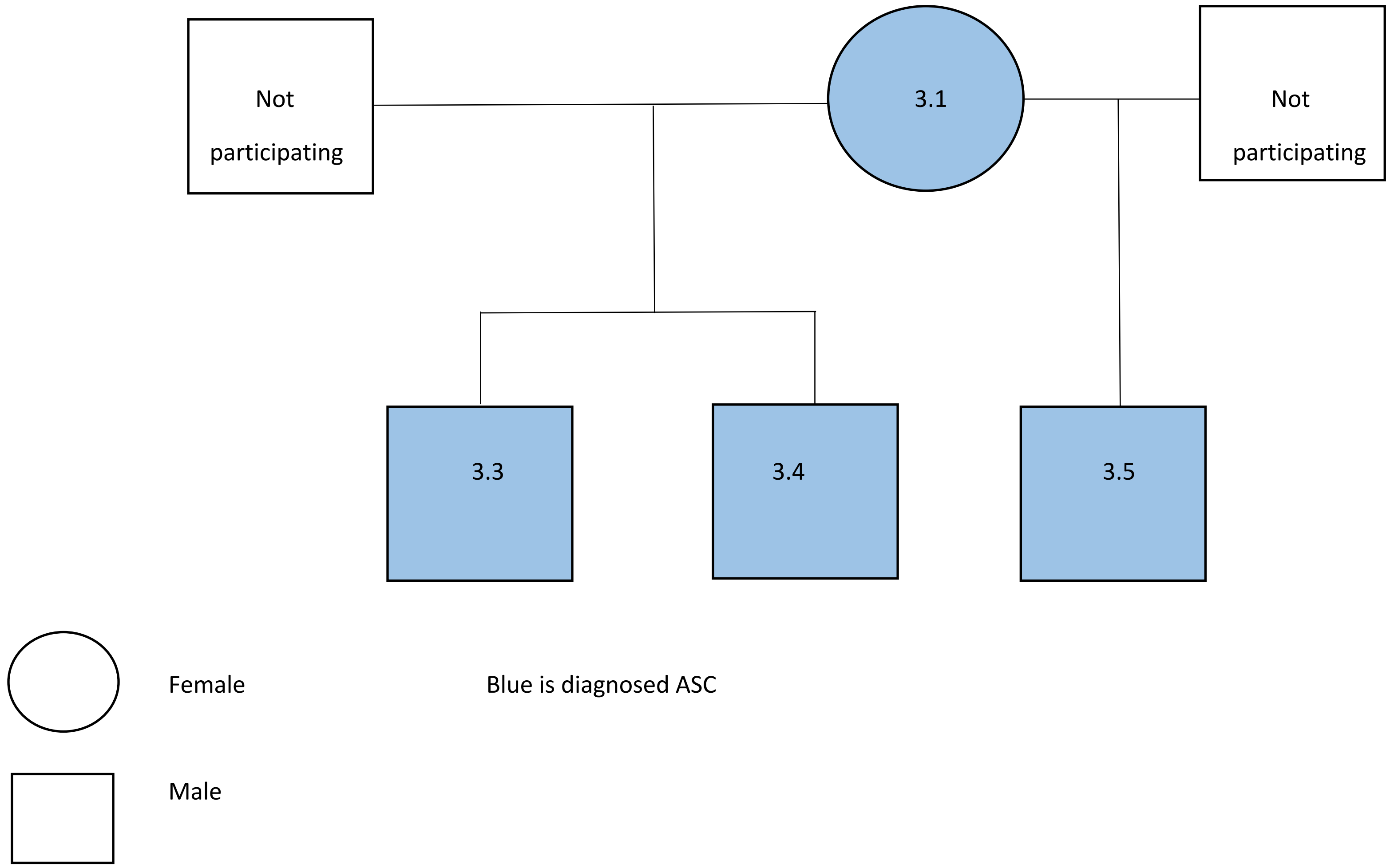
Not participating
3.1
Not participating
3.3
3.4
3.5
Female
Male
Blue is diagnosed ASC

Family 4

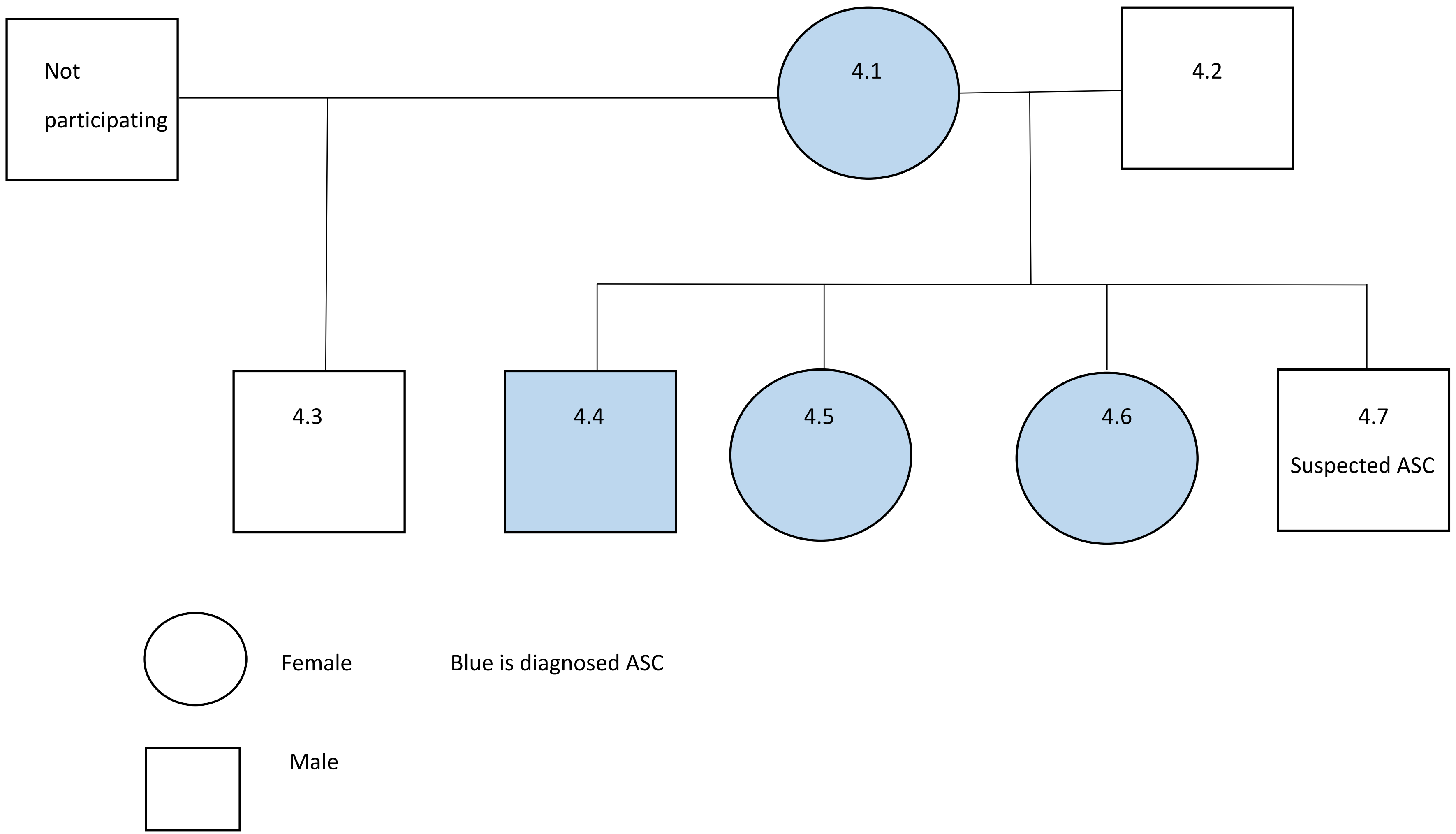
Not
participating
4.1
4.2
4.3
4.4
4.5
4.6
4.7
Suspected ASC
Female
Blue is diagnosed ASC
Male

Family 7

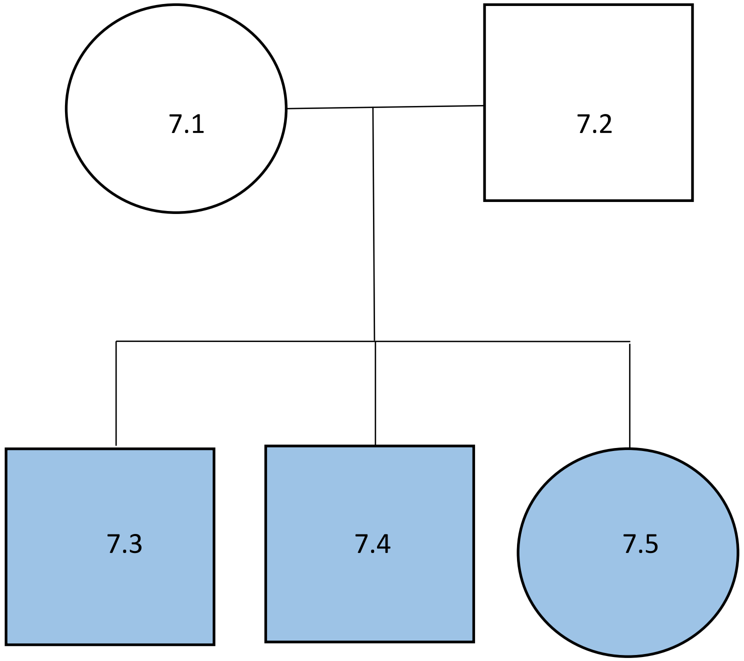

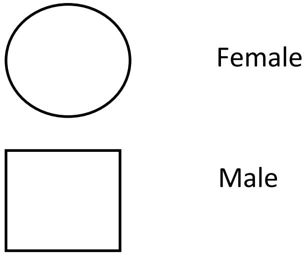

Blue is diagnosed ASC

Family 8

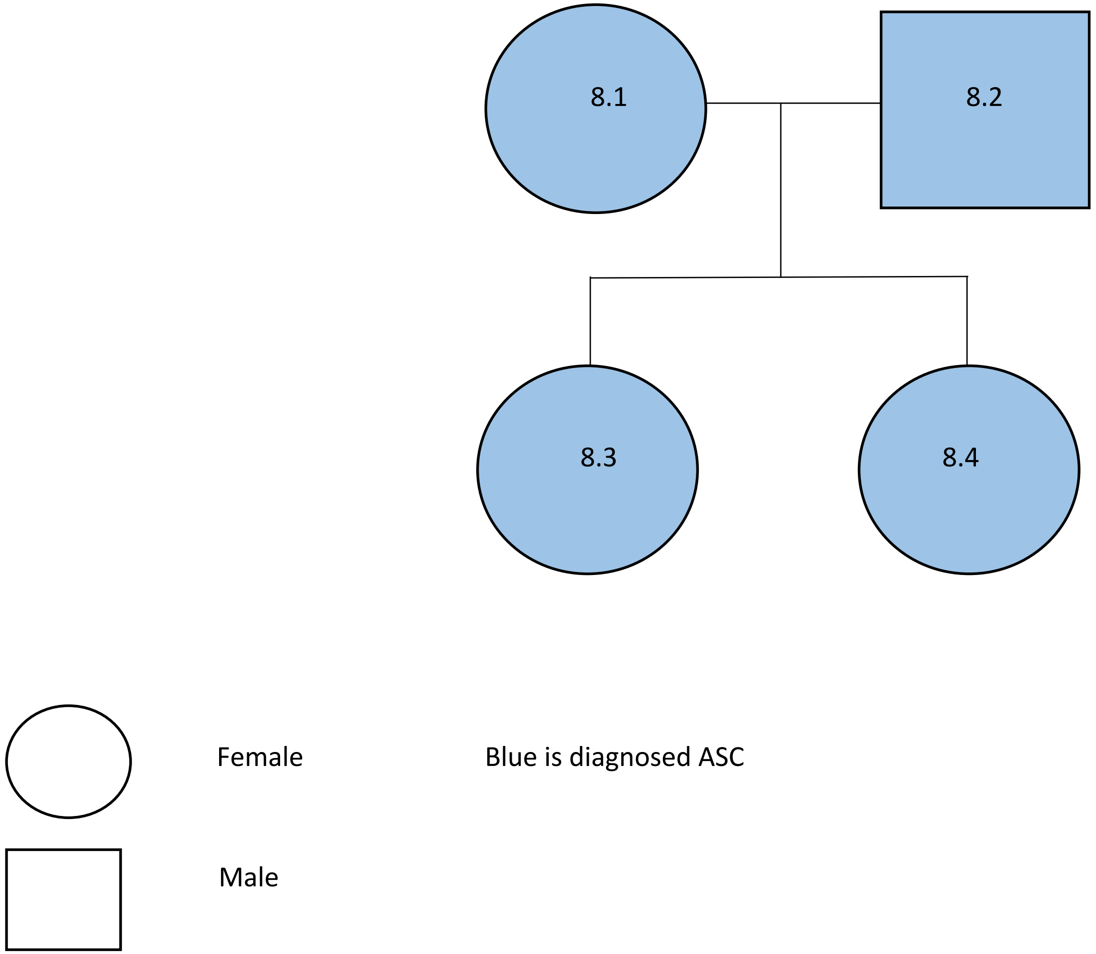

Family 10

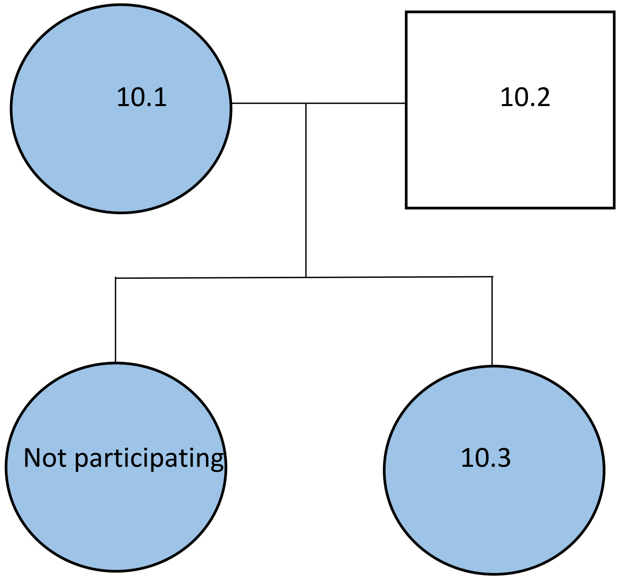
10.1
10.2
Not participating
10.3

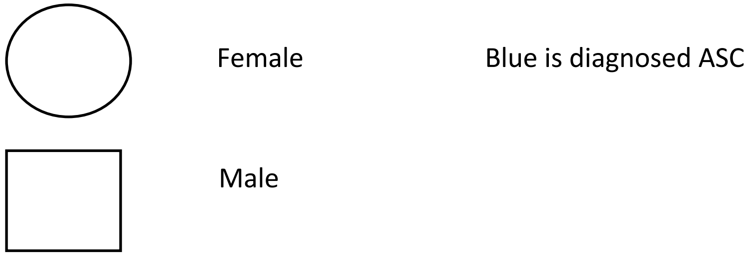
Female
Male
Blue is diagnosed ASC

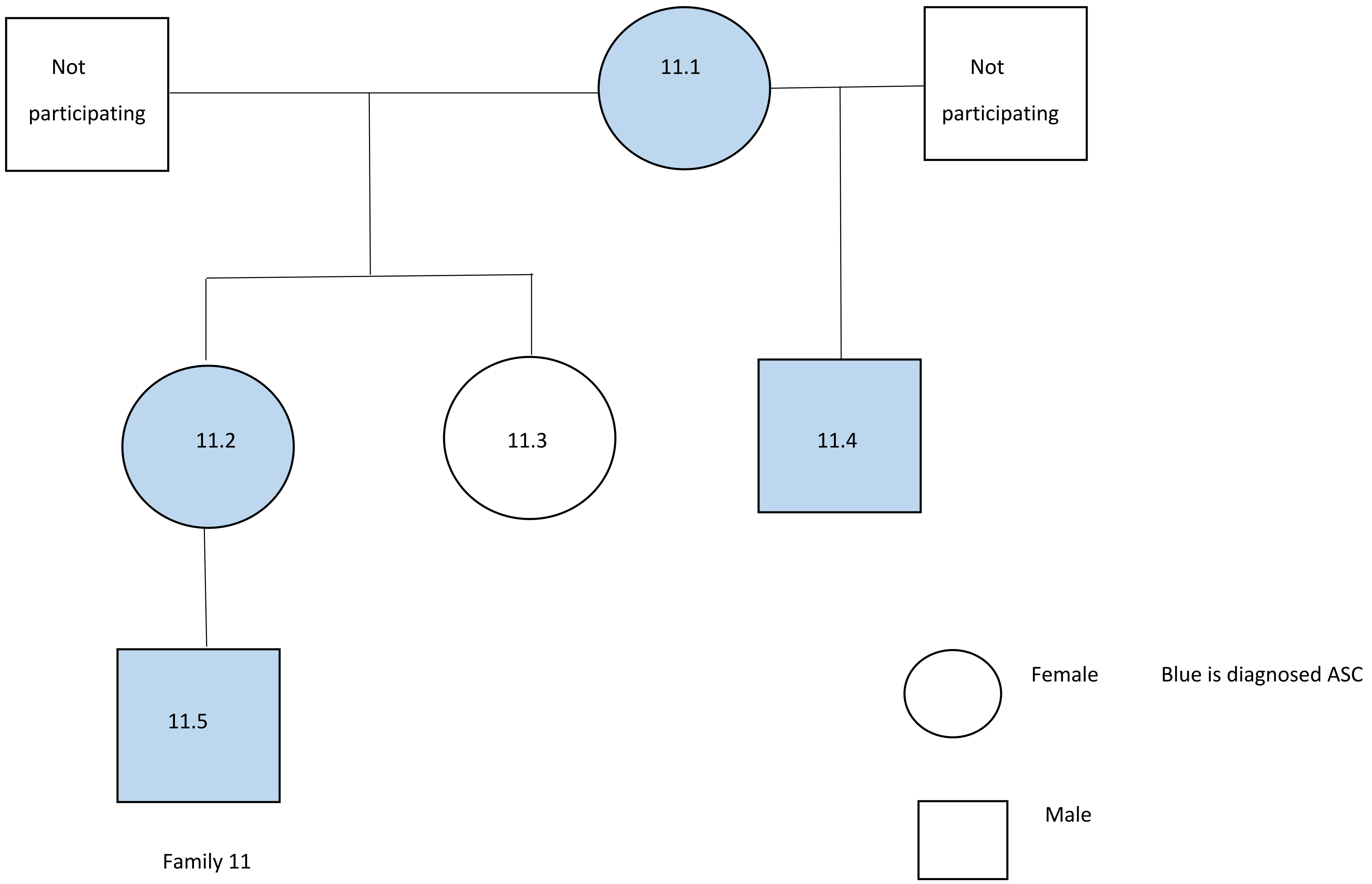
Not participating
11.1
Not participating
11.2
11.3
11.4
11.5
Female
Blue is diagnosed ASC
Male

Family 11

Family 12

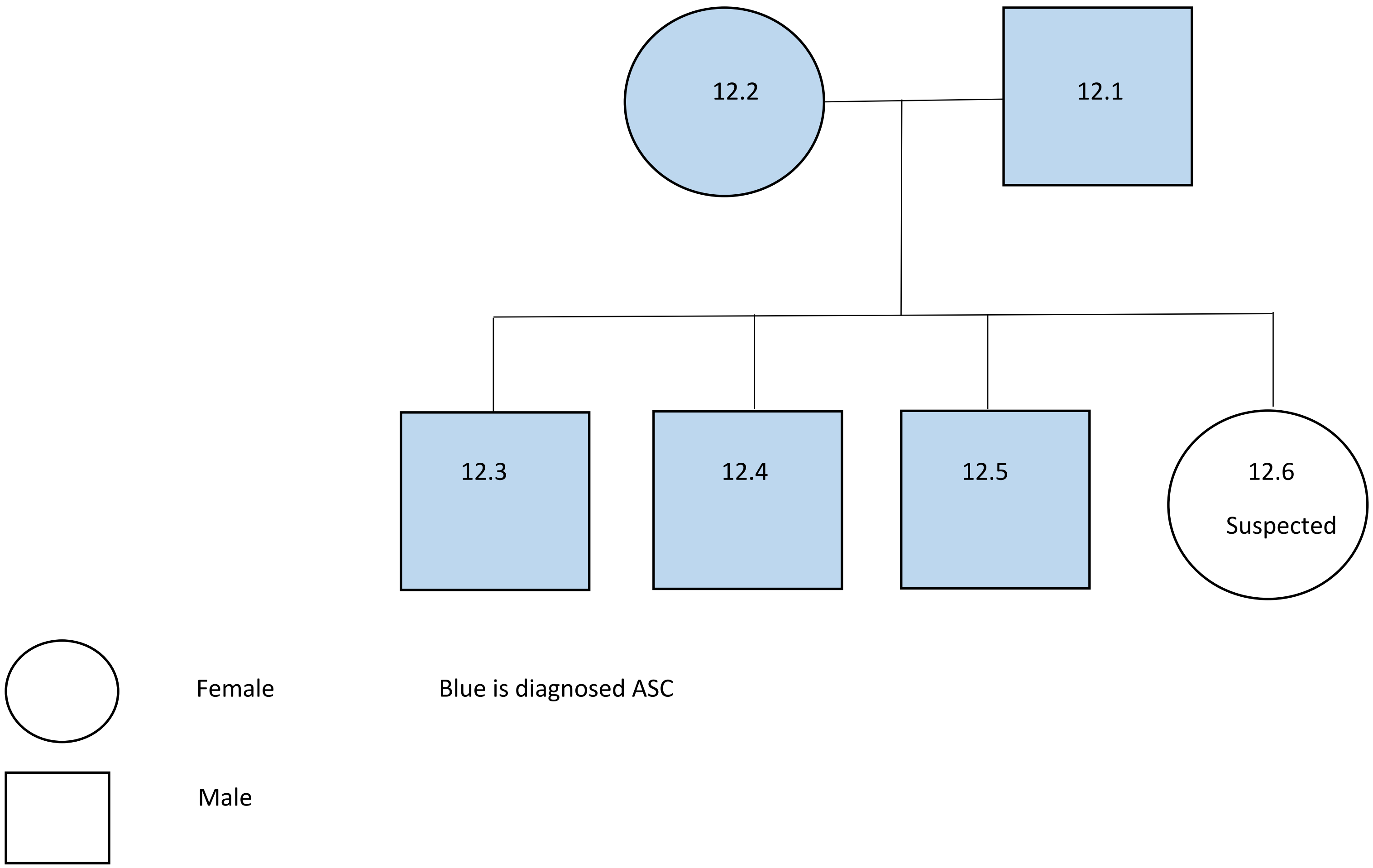
12.2
12.1
12.3
12.4
12.5
12.6
Suspected
Female
Male
Blue is diagnosed ASC

Famly 14

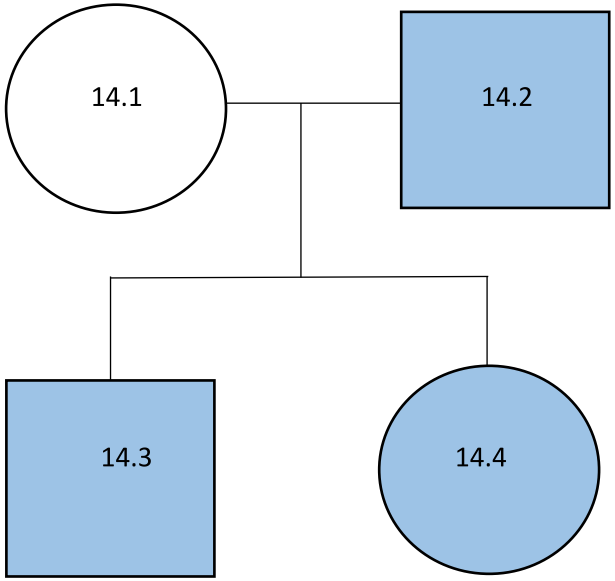

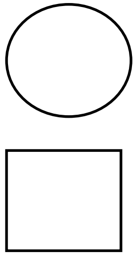

Female

Male

Blue is diagnosed ASC

Famly 15

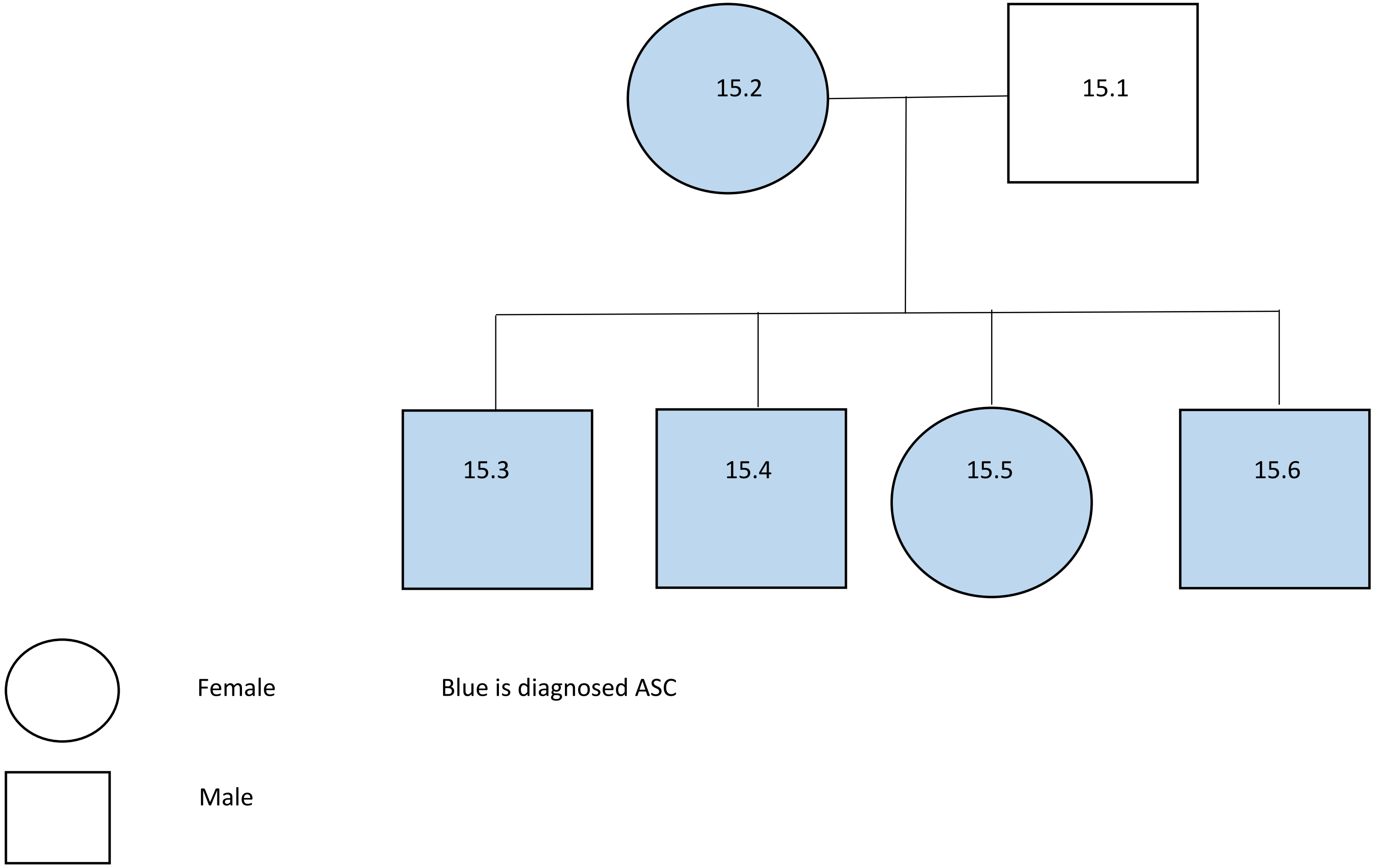

Family 16

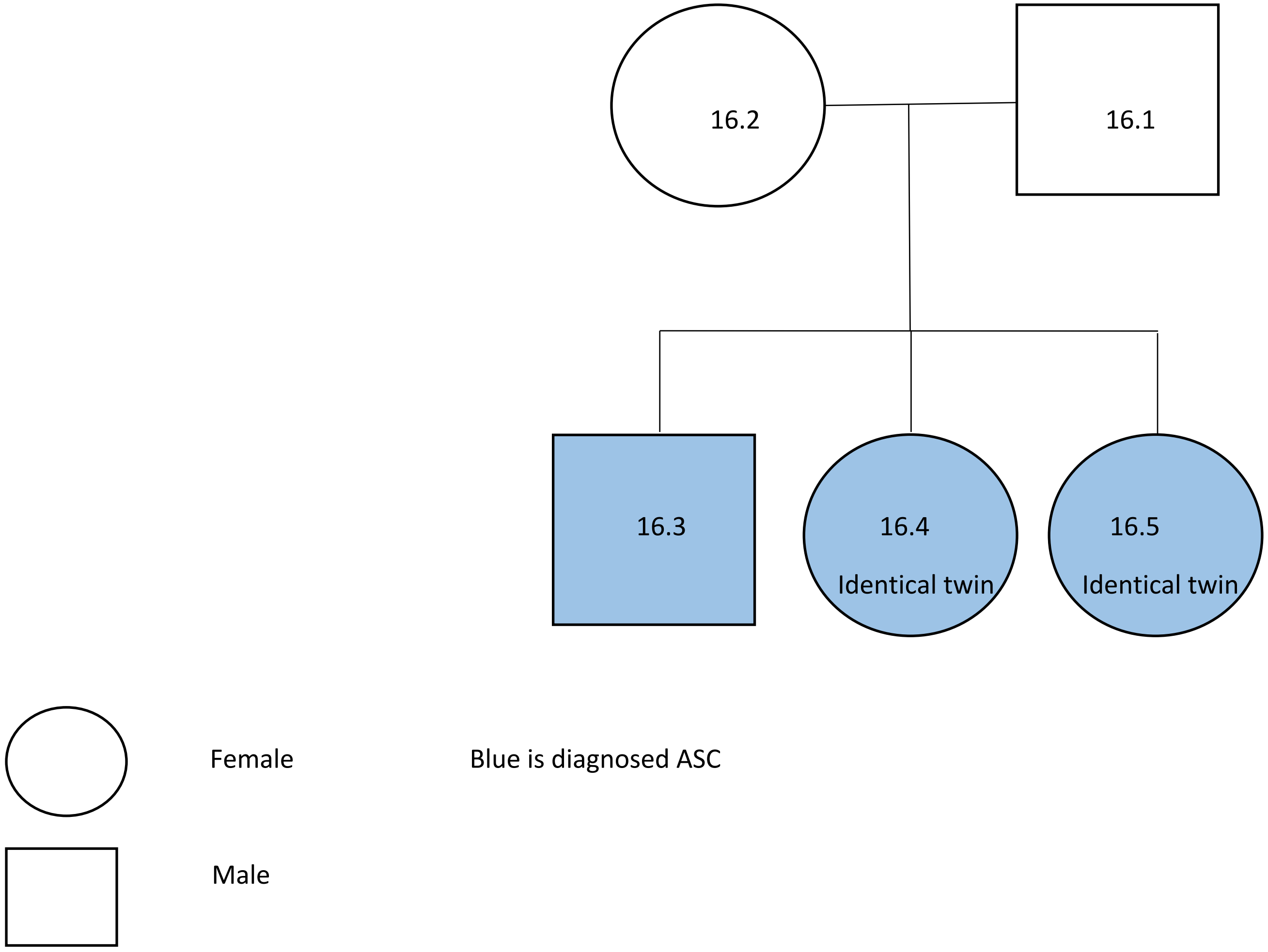
16.2
16.1
16.3
16.4
Identical twin
16.5
Identical twin
Female
Male
Blue is diagnosed ASC

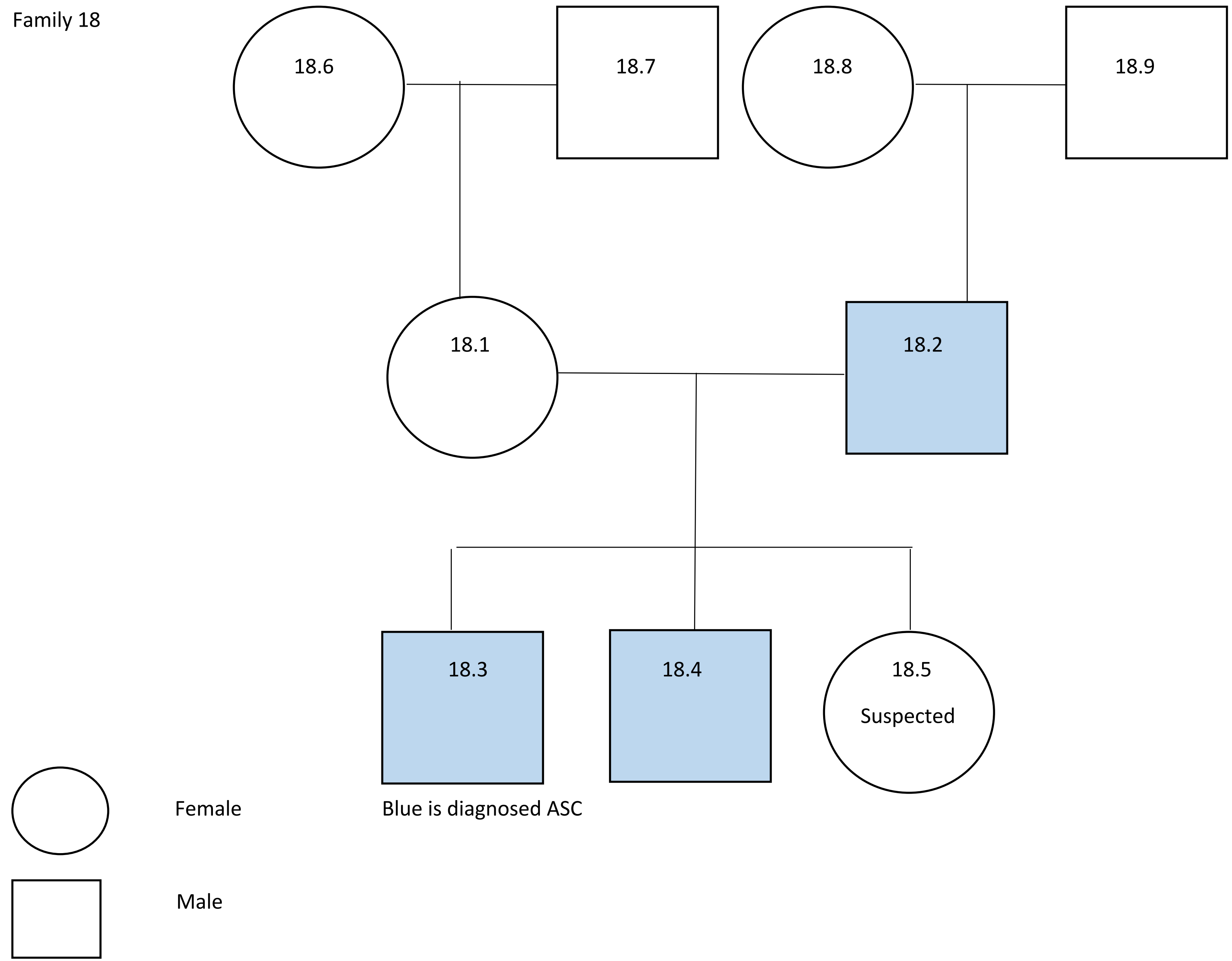
Family 18
18.6
18.7
18.8
18.9
18.1
18.2
18.3
18.4
18.5
Suspected
Blue is diagnosed ASC
Female
Male

Family 19

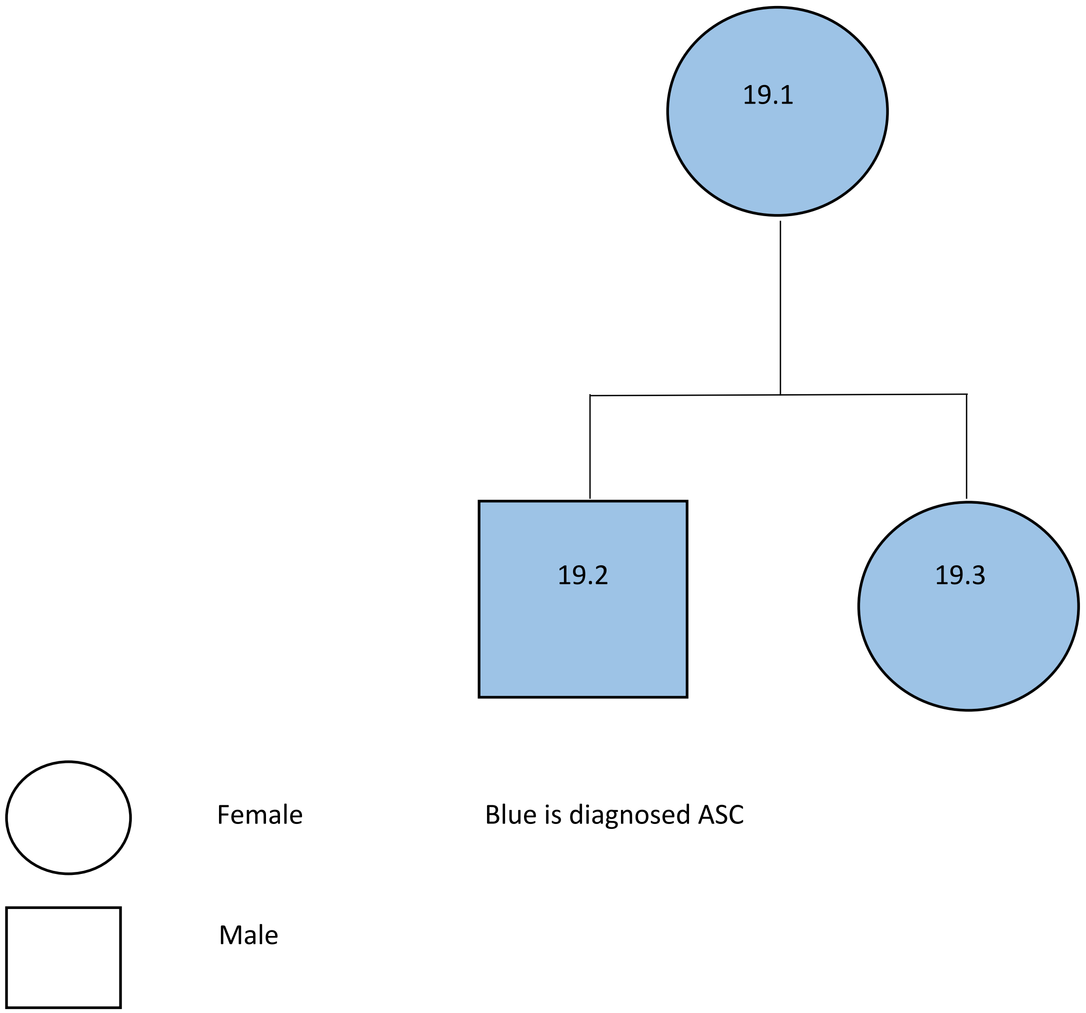

Family 20

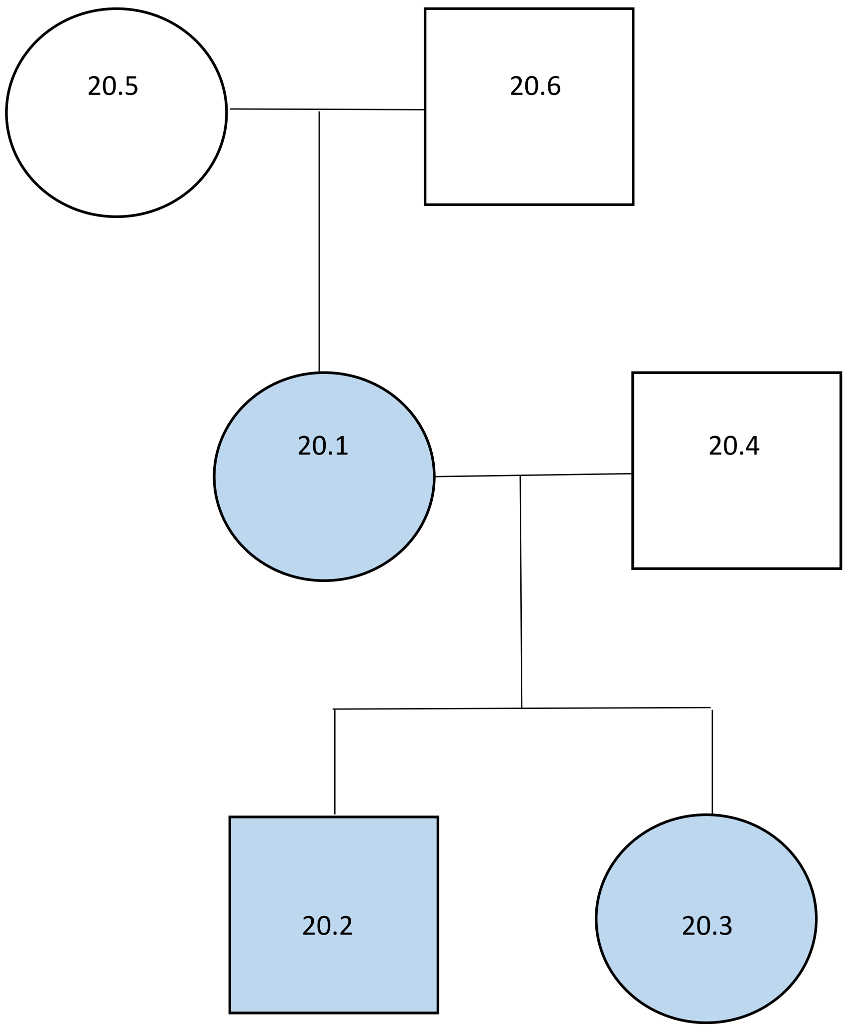

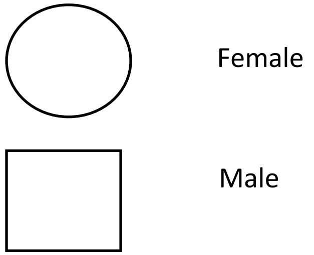

Blue is diagnosed ASC

Family 21

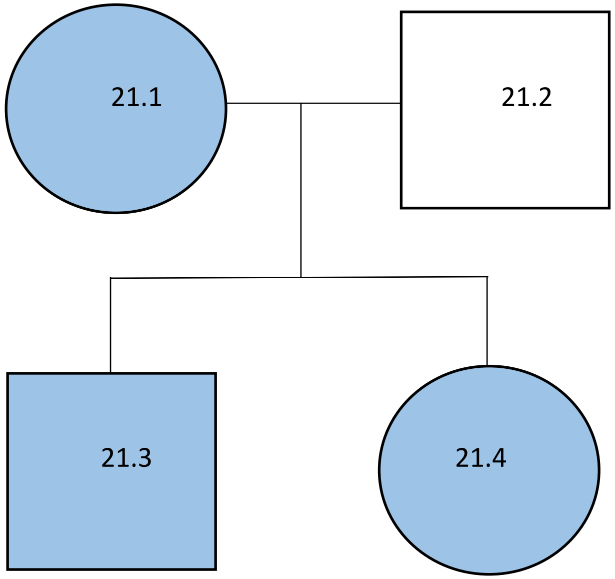

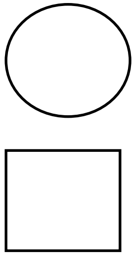

Female

Male

Blue is diagnosed ASC

Family 22

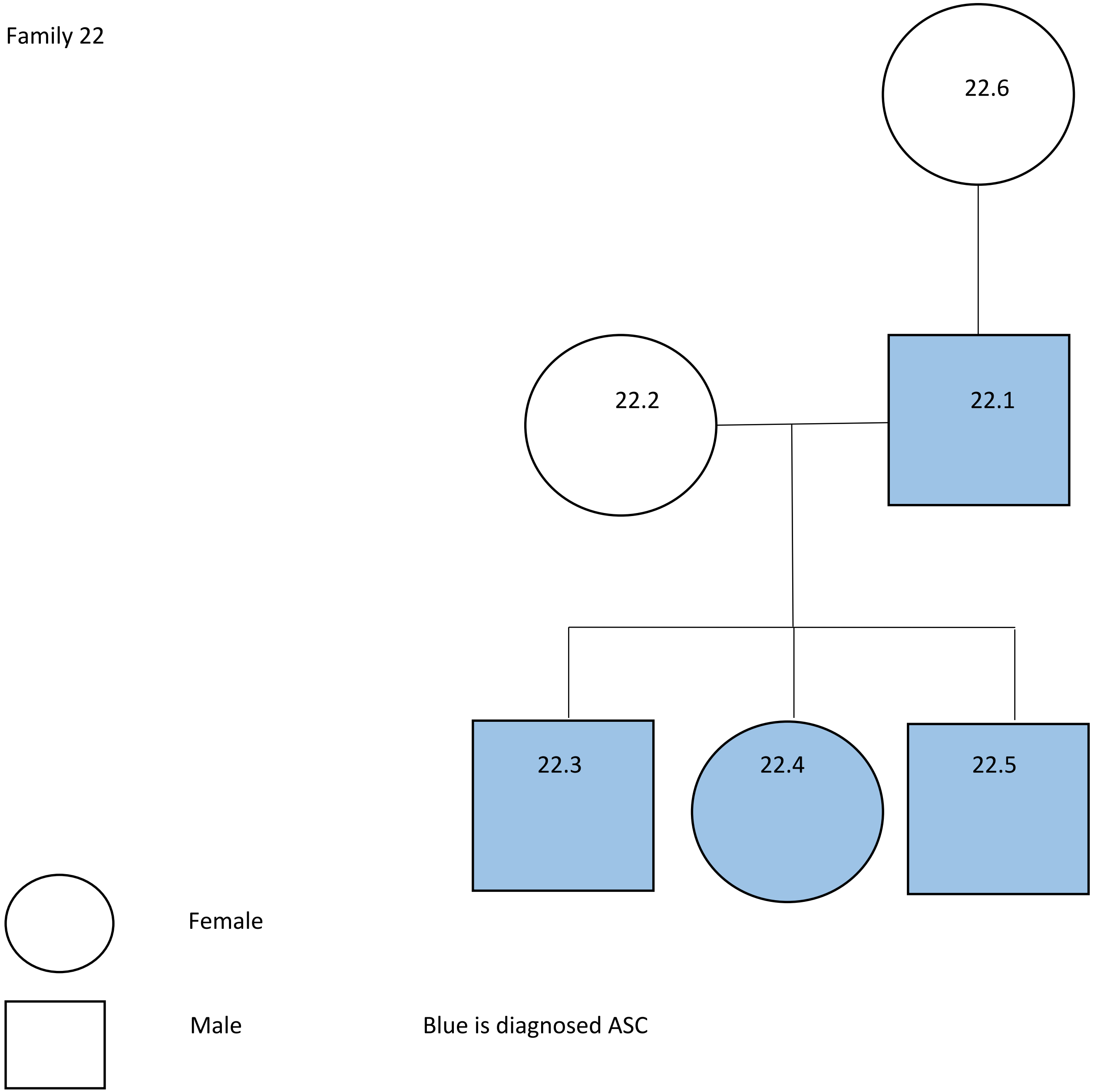

Family 25

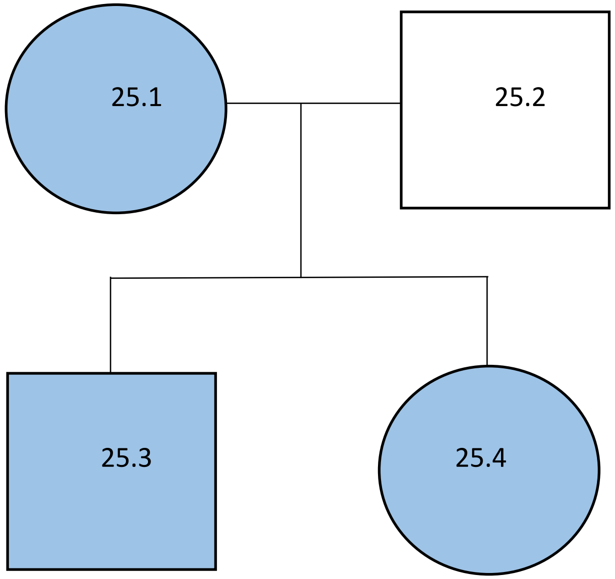

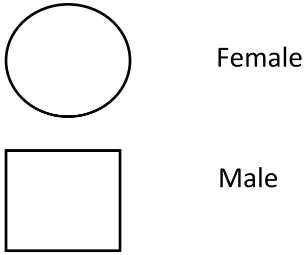

Blue is diagnosed ASC

Family 26

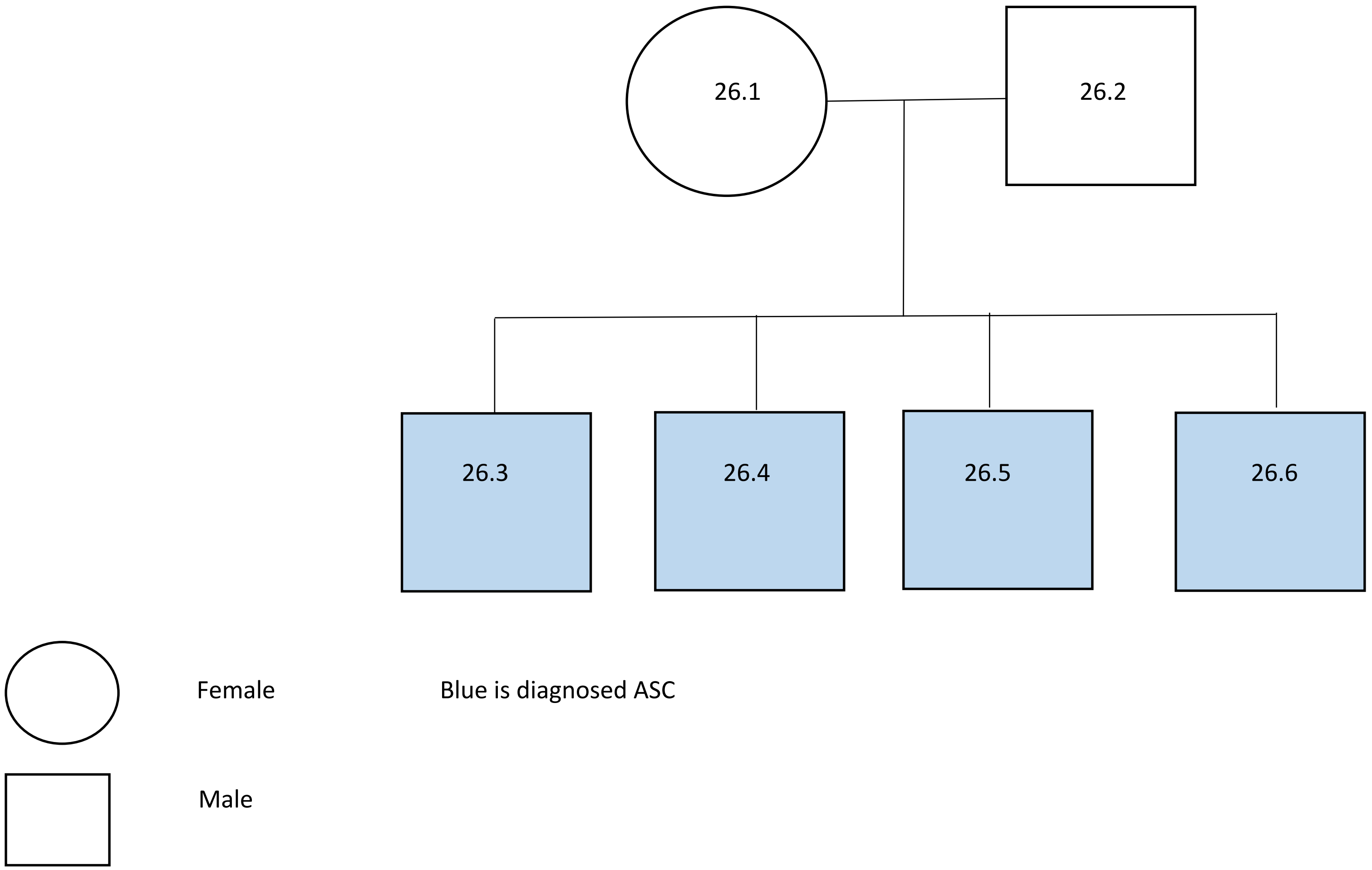

Family 28

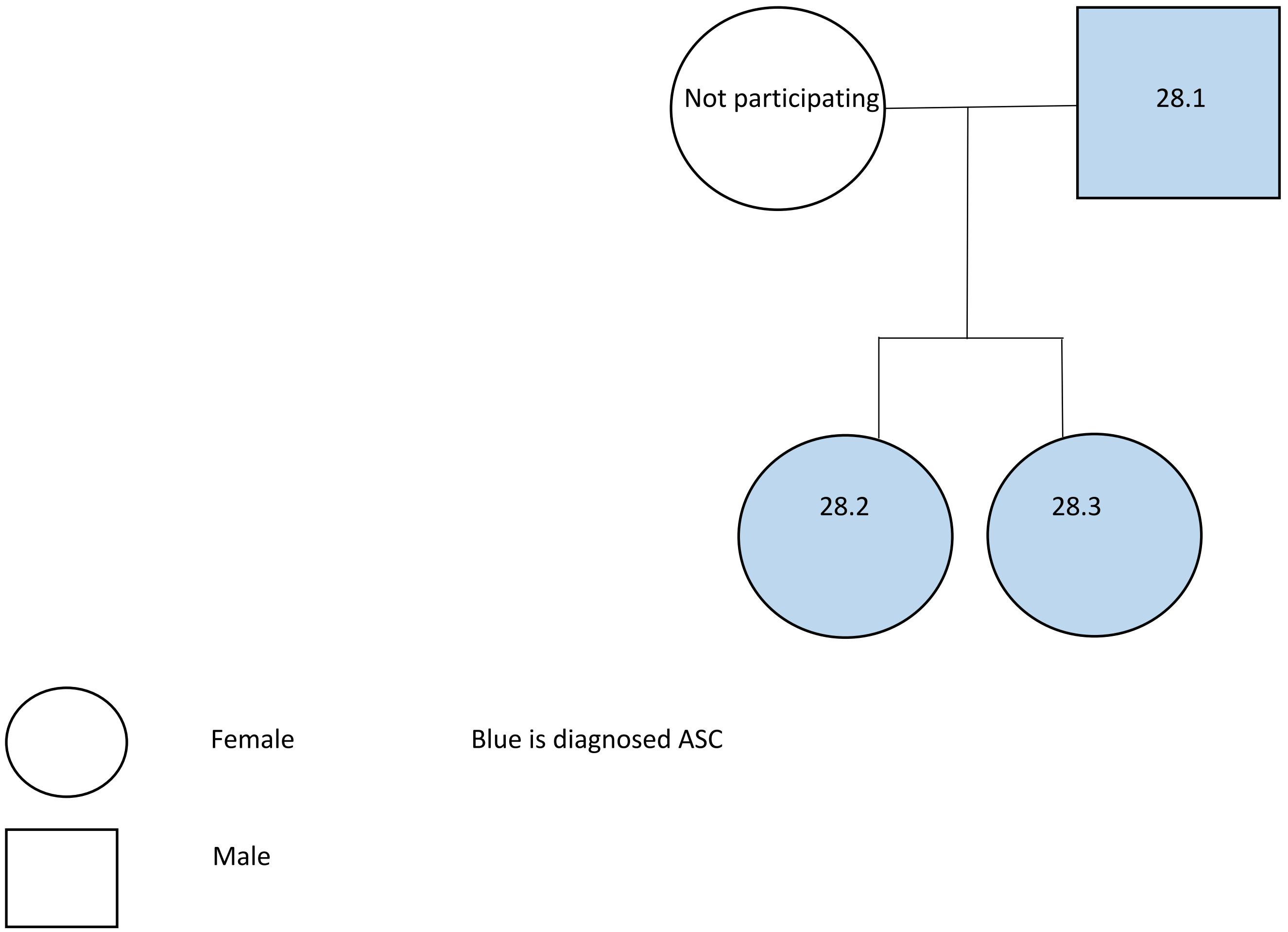
Not participating
28.1
28.2
28.3
Female
Male
Blue is diagnosed ASC

Family 29

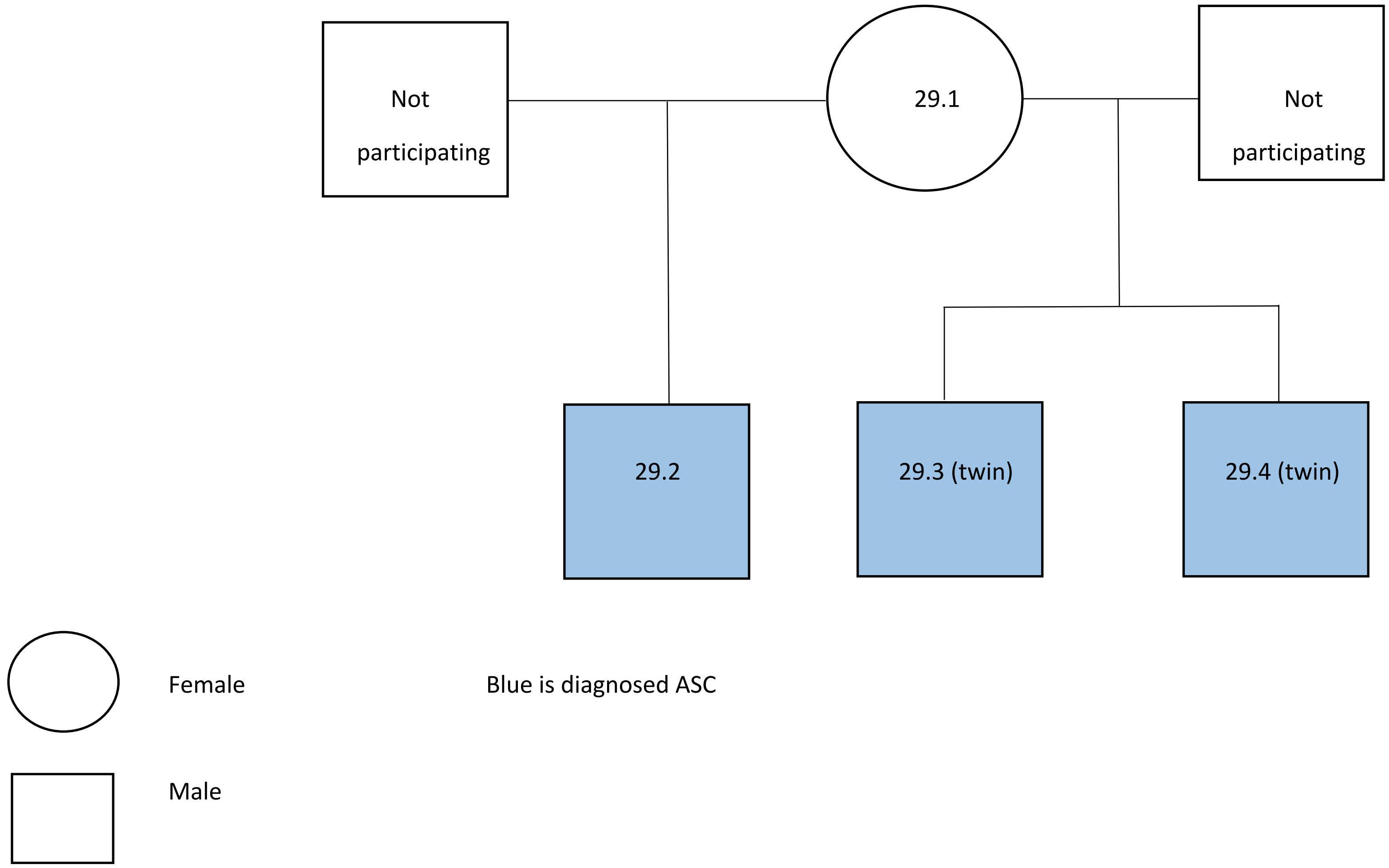
Not participating
29.1
Not participating
29.2
29.3 (twin)
29.4 (twin)
Female
Blue is diagnosed ASC
Male
