# Supplementary figures and images for: Identifying rare genetic variants in 21 highly multiplex autism families: the role of diagnosis and autistic traits
Source: Mol Psychiatry. 2023 Jan 26;28(5):2148–57. doi: 10.1038/s41380-022-01938-4 (PMC10575770; doi:10.1038/s41380-022-01938-4)

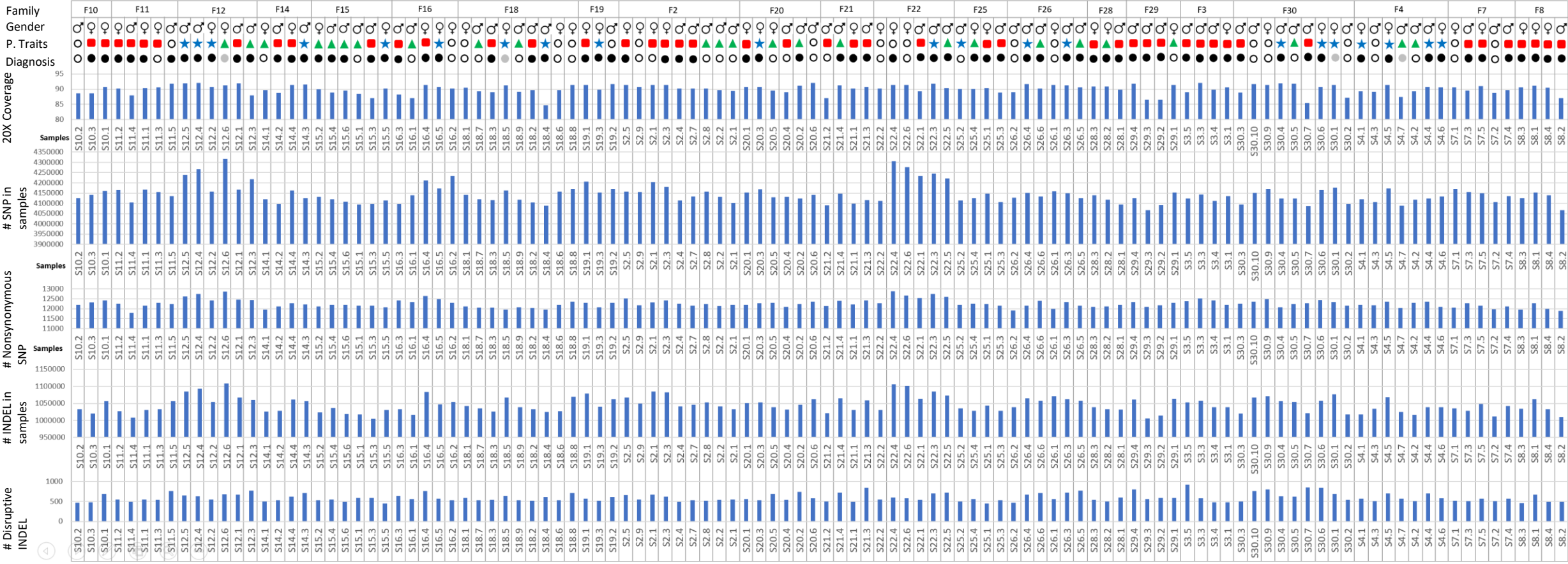

Supplement: Supplementary file 3 — Supplementary Fig. 2 [file 41380_2022_1938_MOESM3_ESM.pdf]
